# p53 and metabolism: from mechanism to therapeutics

**DOI:** 10.18632/oncotarget.25267

**Published:** 2018-05-04

**Authors:** Fernando M. Simabuco, Mirian G. Morale, Isadora C.B. Pavan, Ana P. Morelli, Fernando R. Silva, Rodrigo E. Tamura

**Affiliations:** ^1^ Laboratory of Functional Properties in Foods, School of Applied Sciences (FCA), Universidade de Campinas (UNICAMP), Limeira, São Paulo, Brazil; ^2^ Center for Translational Investigation in Oncology/LIM24, Instituto do Câncer do Estado de São Paulo (ICESP), São Paulo, Brazil; ^3^ Department of Radiology and Oncology, Faculdade de Medicina, Universidade de São Paulo, São Paulo, Brazil

**Keywords:** metabolism, p53, mutant p53, chemotherapy, drug resistance

## Abstract

The tumor cell changes itself and its microenvironment to adapt to different situations, including action of drugs and other agents targeting tumor control. Therefore, metabolism plays an important role in the activation of survival mechanisms to keep the cell proliferative potential. The Warburg effect directs the cellular metabolism towards an aerobic glycolytic pathway, despite the fact that it generates less adenosine triphosphate than oxidative phosphorylation; because it creates the building blocks necessary for cell proliferation. The transcription factor p53 is the master tumor suppressor; it binds to more than 4,000 sites in the genome and regulates the expression of more than 500 genes. Among these genes are important regulators of metabolism, affecting glucose, lipids and amino acids metabolism, oxidative phosphorylation, reactive oxygen species (ROS) generation and growth factors signaling. Wild-type and mutant p53 may have opposing effects in the expression of these metabolic genes. Therefore, depending on the p53 status of the cell, drugs that target metabolism may have different outcomes and metabolism may modulate drug resistance. Conversely, induction of p53 expression may regulate differently the tumor cell metabolism, inducing senescence, autophagy and apoptosis, which are dependent on the regulation of the PI3K/AKT/mTOR pathway and/or ROS induction. The interplay between p53 and metabolism is essential in the decision of cell fate and for cancer therapeutics.

## CANCER METABOLISM

Despite diagnostic and therapeutic advances in the last decades, cancer is still one of the deadliest diseases around the world. The lack of a cure for cancer reflects the complexity of its molecular bases, as illustrated by the hallmarks of cancer since 2000 [1], and updated in 2011 [2]. Warburg effect, also called aerobic glycolysis, known since the 1920s [3], is by definition the shift presented by tumor cells from the complete oxidation of glucose to an incomplete oxidation to lactate, even in the presence of oxygen. This observation may be considered one of the hallmarks of cancer and shows one of the alterations related to metabolism presented by cancer cells [1, 4].

### The Warburg effect and its consequences

Healthy cells in the presence of oxygen usually convert glucose to pyruvate, then acetyl-CoA, which in turn is completely oxidized in the TCA cycle inside the mitochondria, generating during this process 32 molecules of ATP per molecule of glucose. The consumption of oxygen is taken in a process called oxidative phosphorylation (OxPhos) inside the mitochondria. Under hypoxic conditions, those cells are able to convert glucose to lactate, generating 2 molecules of ATP per molecule of glucose.

Cancer cells present an altered behavior. Even in the presence of oxygen, these cells oxidize glucose to lactate, in a process that is several times less efficient to generate ATP and therefore consumes more glucose. This apparent paradox is explained in part by several other alterations in metabolism that occur besides the Warburg effect.

The main consequence of the Warburg effect is the increased consumption of glucose in cancer cells. This property was initially observed by Warburg's experiments and it is now the theoretical basis for diagnostic purposes. The administration of [18F] fluoro-2-deoxy-glucose (FDG) in patients and the analysis by PET (Positron Emission Tomography) scan may reveal sites in the body that greatly incorporate glucose and thus may contain tumor cells. This technique, called FDG-PET, has been widely used to search for different types of cancers and is one of the most elegant proofs of the Warburg effect [5].

**Figure 1:**
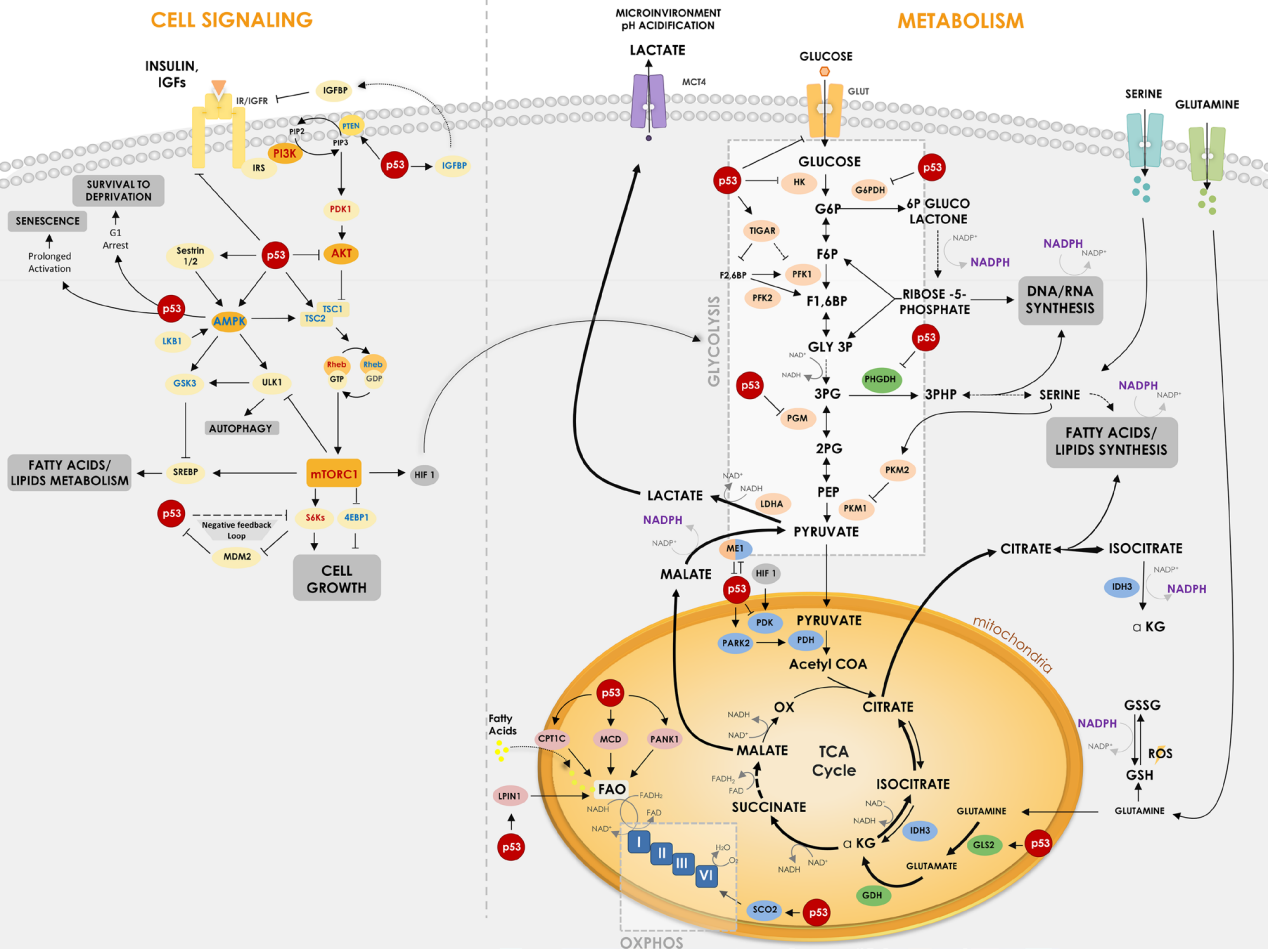
P53 regulates critical points in cell signaling and metabolism p53 is involved in different critical points of metabolism control and cell signaling, being able to regulate key proteins and enzymes in cellular response to cancer. The Figure shows how p53 suppresses glycolysis and increases OxPhos (oxidative phosphorylation) while it regulates IGF signaling pathway. p53 regulates glucose uptake by some glucose transporters (GLUT1, GLUT3 and GLUT4) and regulates direct and indirectly enzymes in its downstream oxidation to pyruvate – HK, TIGAR, PFK1/2, G6PDH, PGM and ME1. p53 is also important for mitochondrial respiration through regulation of MCD, LPIN1, PANK1, CPT1C and SCO2. Furthermore, p53 regulates enzymes involved in amino acids metabolism such as PHGDH, which participates in serine metabolism derived from the 3PG, and GLS2, which catalyzes the hydrolysis of glutamine to glutamate, increasing TCA cycle flux. In cell signaling transduction, p53 reveals a crucial activity in suppressing downstream proteins of the IGF pathway, such as AKT, AMPK and S6Ks, modulating processes such as cell growth, autophagy and lipid metabolism. In cell signaling, proteins with positive relation with cancer are shown in red and negative relation in blue. Regular arrows represent activation and blunt arrows inhibition. Thicker arrows indicate increased reactions in cancer when compared to thin arrows.

The increased consumption of glucose and the preference to oxidize it to lactate is actually observed in any proliferating cell [4, 6, 7]. The main reason for that is a detour from an energy production state to a biosynthetic state, where it is more important for the cell to acquire the building blocks of its structure, such as nucleotides, amino acids, lipids and NADPH - an important tool for biosynthesis - than ATP production. Accordingly, part of the glucose consumed through the Warburg effect enters the Pentose Phosphate Pathway (PPP), generating ribose-5-phosphate and NADPH (Figure 1), which are greatly demanded in proliferating cells that need to duplicate their DNA mass and actively synthesize RNA [4, 6, 7].

The second most evident consequence of Warburg effect is the increased levels of lactate secretion. The secretion of lactate is important to maintain pH homeostasis inside the cell, but it also plays roles for cancer progression outside the cell. It has been demonstrated that extracellular environment acidosis precedes angiogenesis, and thus lactate may stimulate angiogenesis in a hypoxia-inducible factor 1 (HIF-1) independent manner [8]. Besides, acidosis of extracellular matrix may increase local invasion of the cancer cells [9] and inhibit infiltration of T lymphocytes [10], thus presenting additional advantages for tumor progression.

A third consequence of the Warburg effect is the reduced usage of the respiratory chain in the mitochondria, due to reduced levels of OxPhos and oxygen consumption. Accordingly, less reactive oxygen species (ROS) are produced by aerobic glycolysis compared to oxidative phosphorylation, which may lead the cancer cells to escape apoptosis and to enhance proliferation [11, 12].

### Beyond Warburg effect

The alterations in metabolism in a cancer cell go beyond the Warburg effect. As it might be expected, the aerobic glycolysis shunt increases the energy demand by the cells, which is supplied by alternative metabolic pathways, like glutamine and fatty acid oxidation through the TCA cycle (Figure 1).

It has long been known that cancer cells and actually any proliferating cell have increased consumption of glutamine [13, 14]. Glutamine can enter the TCA cycle and be oxidized to lactate, which contributes for media acidification and also NAPDH production, through the cytoplasmic malic enzyme 1 (ME1), as seen in Figure 1 [15]. Besides, glutamine metabolism is an important source of glutathione (GSH), which is used by the cell to control oxidative stress [16]. NADPH is used in this case to regenerate (reduce) the oxidized form of GSH - named GSSG -back to GSH, after ROS clearance.

Dividing cells, including cancer cells, have also an increased demand for lipids, since increase in size and number demand phospholipids to form their plasma membrane and their inner membranes of organelles. The increased consumption of glutamine is an important source of carbon to generate acetyl-CoA, precursor for lipid synthesis, using alternative routes of the TCA cycle, as seen in Figure 1 [17]. Besides, lipid biosynthesis has a great demand for NADPH, which is supplied by PPP, ME1 and the cytoplasmic isoform 1 of isocitrate dehydrogenase (IDH1).

Glutamine metabolism through TCA cycle is also an important source of amino acids, which is a great demand in dividing cells since protein synthesis is increased. Alpha-ketoglutarate, oxaloacetate and pyruvate are precursors of several amino acids and thus their flux through TCA cycle is active in cancer cells. Nevertheless, TCA complete cycling is altered in cancer cells (see arrows patterns in Figure 1).

Another enzyme, that has been characterized as altered in tumors, is the pyruvate kinase (PK), the last step of the glycolytic pathway, responsible for phosphoenolpyruvate (PEP) conversion in pyruvate, generating ATP (Figure 1). The PKM2 isoform is a splicing variant of this enzyme that is found in proliferating cells, in embryonic tissues and in several types of cancer cells [18–20]. PKM2 has diminished activity compared to PKM1, thus slowing down the last reaction of the glycolytic pathway, which may look disadvantageous, but indeed is beneficial to cancer cells, since decreasing the speed of the last steps of glycolysis increases the flux of glycolysis intermediates to PPP, thus generating more NADPH and precursors for nucleotide biogenesis like ribose-5-phosphate [20–22]. Besides, accumulation of these intermediates, as 3-phosphoglycerate (3PG), favors serine/glycine *de novo* synthesis pathway, also important for nucleotide synthesis.

Serine/glycine metabolism is indeed another altered pathway in cancer (Figure 1). Serine and glycine are known precursors of phospholipids, nucleotides and GSH, which are important for cell growth and proliferation and redox control, as already discussed. It is well described that cancer cells have increased consumption of serine [23, 24]. Besides, the first enzyme of the serine pathway, called phosphoglycerate dehydrogenase (PHGDH), that converts 3-phosphoglycerate (3PG) to 3-phosphohydroxypyruvate (3PHP), is overexpressed in several types of tumors [25, 26]. Interestingly, serine, together with fructose-1,6-bisphosphate (F16BP), are allosteric activators of PKM2, meaning that serine starvation inhibits the flux through the glycolytic pathway, accumulating intermediates for *de novo* serine synthesis [22, 27] (Figure 1).

Proline is another amino acid that seems to have a role in cancer. Proline is produced either by glutamate or by arginine-derived ornithine, where pyrroline-5-carboxylate reductase (PYCR1) is one of the main enzymes of proline biosynthesis. Evidences suggest that PYCR1 is upregulated in several types of tumors [28]. Conversely, proline oxidase or dehydrogenase or p53-induced gene 6 (POX, PRODH, PIG6) expression, which participates in proline degradation, inhibits tumor growth inducing cell cycle arrest [29].

Overall, the alterations related to metabolism in cancer implicate complex relationships between metabolic pathways and regulatory networks, involving several metabolic intermediates and expanding Warburg's first observations. As it will be discussed, the tumor suppressor p53 plays a pivotal role in regulating the expression and function of several of metabolic genes.

## REGULATION OF METABOLISM BY P53

P53, encoded by the gene *TP53*, was first described as a cellular protein that interacts with an oncogenic viral protein [30–33]. The initial observation indicated that overexpression of p53 induced cell transformation and p53 was therefore considered an oncogene [34–36]. Nonetheless in the next few years other reports indicated an opposite effect of p53 and the cloned wild-type p53 was devoid of oncogenic activity [37]. It soon became clear that mutant forms of p53 were responsible for the early oncogenic observations. Indeed p53 is the most frequently mutated gene in cancer, reaching a prevalence of about 95% in serous ovarian cancer and with a mean frequency of 42% [38]. Gene deletion or truncation are frequently observed in tumor suppressors, like BRCA1 and Rb that show weak or no expression of the proteins, however, the most frequent alteration in p53 gene are missense mutations [39]. Instead of loss of expression, there´s an altered p53 protein with a “gain of function” (GOF) mutation that is tumorigenic and favors cell transformation.

Even though induction of apoptosis and cell cycle arrest are the hallmarks of p53 activity, it's role in metabolism have been recognized and p53 is acknowledged as a general stress sensor, not only important to inhibit cancer progression, but also to respond during viral infection, starvation or oxidative stress, reducing cell proliferation, altering cellular metabolism and inhibiting survival [40]. As a general stress sensor, the ability to circumvent p53 is essential for the progression of DNA mutation, metabolism alteration, oxygen deprivation and viral infection.

**Figure 2:**
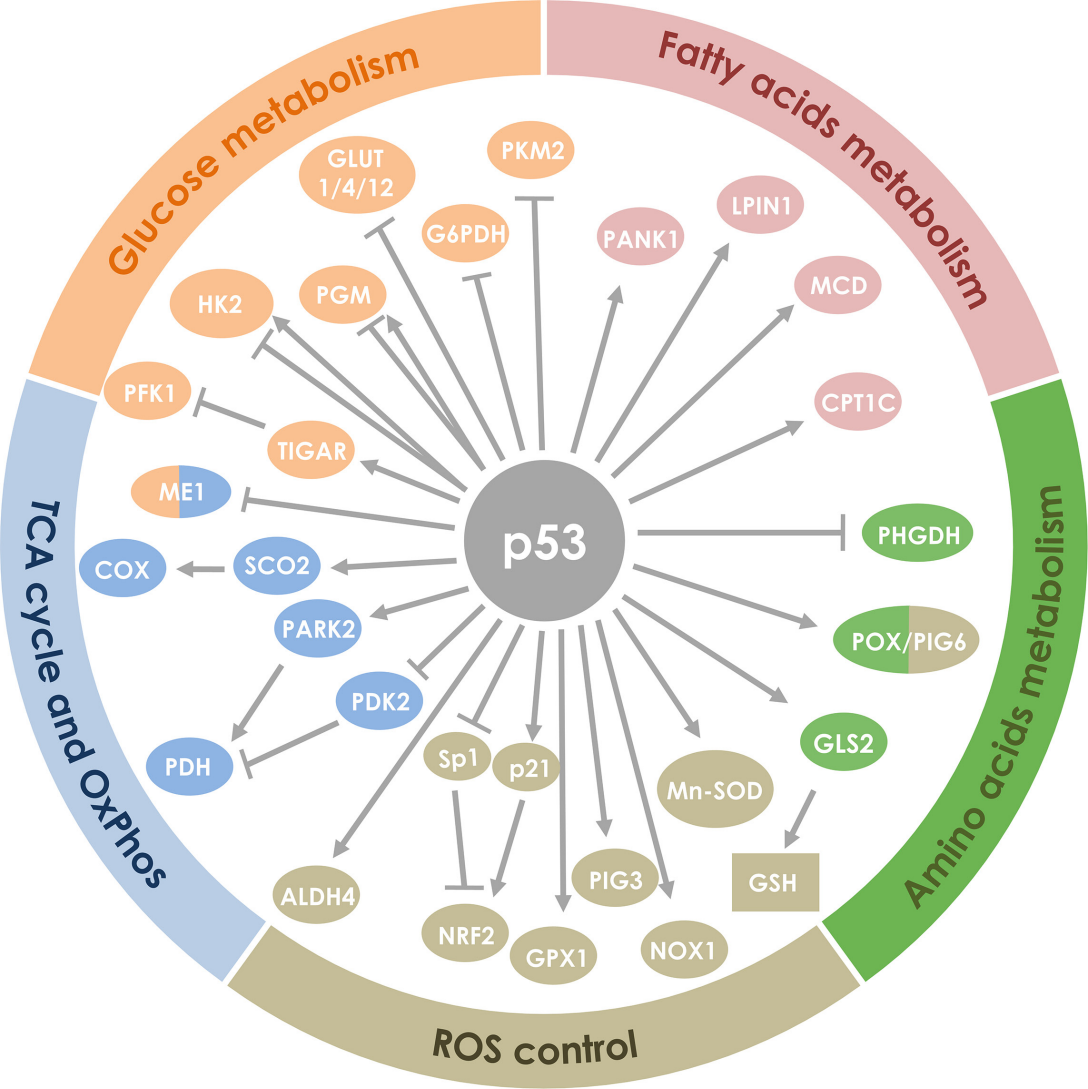
Regulation of proteins involved in cell metabolism by p53 Proteins (indicated by circles) and other molecules (indicated by squares) were grouped according to their involvement in the metabolism of glucose, amino acids and fatty acids, ROS regulation, TCA cycle and the oxidative phosphorylation. Regular arrows represent activation and blunt arrows inhibition.

Wild-type p53 is upregulated after DNA damage and induces cell cycle arrest and apoptosis and is considered the “guardian of the genome” [41]. p53 can directly bind to proteins involved in apoptotic signaling, however, its main action mechanism is through transcriptional regulation of genes involved in cell cycle, induction of apoptosis, angiogenesis, autophagy, immunomodulation and several other processes that can hamper cancer progression. There are in the human genome more than 4,000 p53 binding sites that regulate the expression of more than 500 genes [42, 43]. From these at least 75 are involved with biosynthesis and metabolism [44]. Figure 1 shows how p53 induces/inhibits key metabolic genes involved in glycolytic pathway, OxPhos, lipid and amino acids metabolism and cell growth. Figure 2 summarizes the p53 key target proteins involved in TCA cycle, ROS control, amino acids, fatty acids and glucose metabolism.

### p53 and glucose metabolism

TP53-induced glycolysis and apoptosis regulator (TIGAR) is one of the best-known proteins regulated by p53 that mediates its action in metabolism. Expression of TIGAR is stimulated early by low levels of p53 and plays a role in ROS inhibition and cell survival allowing time for DNA repair mechanisms. However, after prolonged stress, its expression is reduced and pro-apoptotic genes are induced instead. TIGAR induction leads to inhibition of the glycolysis enzyme Phosphofructokinase (PFK-1) and redirection of glucose to PPP, thus increasing NADPH and other intermediates, which are essential DNA precursors used during DNA repair. TIGAR also degrades fructose-2,6-bisphosphate (F26BP), a strong allosteric activator of PFK-1 [45] (Figure 1). However, this redirection to PPP may be abrogated by p53 direct inactivation of glucose 6-phosphate dehydrogenase (G6PDH); mutant p53 lacks this ability, thus increasing PPP flux and glucose consumption (Figure 1) [46].

A study has demonstrated that the loss of both p53 and PTEN in prostate cancer cells is responsible for an increase in expression of HK2, contributing for the aerobic glycolysis [47]. Phosphoglycerate mutase 1 (PGAM1 or PGM1), the enzyme that converts 3-phosphoglycerate (3PG) to 2-phosphoglycerate (2PG), is also downregulated by p53 (Figure 1) [48]. It is known that p53 loss increases PGAM1 expression and activity, hence increasing glycolysis and biosynthesis for tumor growth [49]. In the muscle, however, p53 seems to be a transcriptional activator of a tissue specific PGAM (PGMA2) [50]. HK2 and PGAM1 are examples of glycolytic enzymes downregulated by p53.

ME1, which converts malate to pyruvate and vice versa, an anaplerotic reaction linking glycolysis to the TCA cycle, has also a relationship with p53. A study has shown that ME1 and ME2 can regulate p53, where their downregulation induce p53 activation and p53 associated senescence but not apoptosis; alternatively they can be regulated by p53, where p53 represses their expression, regulating NADPH production and glutamine metabolism (Figure 1) [51].

Finally, it is known that glucose receptors like GLUT1 and GLUT4 may be downregulated by wild-type p53, while mutated forms of p53 induce cancer cells to increase expression of those receptors and consequently to increase the glucose consumption associated with Warburg effect [52]. GLUT3 has also been found to become indirectly down-regulated by p53 through inhibition of IKK [53] and, more recently, it has been shown that p53 is also able to bind and to repress the GLUT12 gene promoter [54]. Overall wild-type p53 acts to reduce glucose intake and aerobic glycolysis (Figure 1).

### p53, TCA and the oxidative phosphorylation

p53 is also able to inhibit pyruvate dehydrogenase kinase 2 (PDK2), a negative regulator of pyruvate dehydrogenase (PDH), thus increasing activity of the later and the flux through TCA cycle (Figure 1) [55]. The increased conversion of pyruvate to acetyl-CoA favors the oxidative phosphorylation and diminishes conversion of pyruvate to lactate, therefore loss of p53 and consequent loss of PDK2 inhibition contributes to the Warburg effect [55]. Conversely, p53 is able to increase expression of Parkin (PARK2), a Parkinson disease associated gene that is able to activate PDH (Figure 1). It has been demonstrated that PARK2 deficiency due to p53 loss increases glycolysis and reduces oxidative phosphorylation, thus contributing to the Warburg effect [56].

Several studies have shown the regulation of oxidative phosphorylation by p53 through synthesis of cytochrome C oxidase 2 protein (SCO2), which induces the cytochrome C oxidase complex (COX) synthesis (Figure 1). COX catalyzes the transfer of electrons to oxygen molecules, in the complex IV of the electron transport chain, a crucial step for OxPhos completion. It has been demonstrated that, in normal cells, wild-type p53 induces SCO2 expression, ensuring increased levels of OxPhos [57]. However, in several types of cancer cells, loss of p53 decreases SCO2 expression and the synthesis of a complete electron transport chain, impairing OxPhos and contributing for the shift to the incomplete oxidation of glucose to lactate [57, 58]. Conversely, SCO2 re-expression in p53-deficient cancer cells is able to rescue oxidative phosphorylation and oxygen consumption levels [58].

### p53 and amino acids metabolism

As already discussed, glutamine is a very important source of carbon, NADPH and glutathione for cancer and any proliferating cells, contributing to redox control. Glutaminase (GLS) is the first enzyme that participates in glutamine metabolism, converting it to glutamate. Glutamate is then converted to alpha-ketoglutarate by glutamate dehydrogenase (GDH). p53 is known to upregulate GLS2, a liver-specific isoform of glutaminase, increasing alpha-ketoglutarate flux through TCA cycle, mitochondria respiration and also redox control by increasing GSH levels (Figure 1) [59, 60]. Therefore, p53 loss in the liver may create an unbalance in the redox homeostasis and increase DNA damage, contributing to cancer progression [60]. Since GLS2 upregulation by p53 increases OxPhos due to increase of carbon flux trough TCA cycle and NADH and FADH_2_ production, the loss of p53 may also decrease OxPhos through GLS2, contributing to the Warburg effect [61].

Phosphoglycerate dehydrogenase (PHGDH), which participates in the serine metabolism derived from the glycolysis intermediate 3-phosphoglycerate (3PG), is also a target of p53, with p53 regulatory elements in its promoter (Figure 1) [62]. It has been recently demonstrated that p53 suppresses PHGDH expression, therefore inhibiting serine biosynthesis, while serine starvation enhances p53-mediated cell death in melanomas, an approach that may have therapeutic applications [63]. Cancer cells have the ability to inhibit glycolysis rapidly under serine starvation, activating the *de novo* serine synthesis. However, cancer cells lacking p53 have increased sensitivity to serine starvation, triggering oxidative stress and inhibiting proliferation [23].

It has also been demonstrated that p53 is able to upregulate, in response to genotoxic damage, the expression of POX, the first enzyme of proline catabolism, regulating the balance between proline and glutamate and their derivate alpha-ketoglutarate [64]. It has been proposed that proline becomes available to cells as a stress substrate of collagen degradation, and might be a signaling molecule for p53 pathway [65].

### p53 and lipids metabolism

Several studies have demonstrated the role of p53 to promote fatty acid oxidation, contributing for cell survival under starvation of nutrients (Figure 1). As examples, p53 is able to activate: carnitine palmitoyltransferase 1C (CPT1C), responsible for fatty acids transportation for oxidation [66]; malonyl CoA decarboxylase (MCD), which converts malonyl-CoA to acetyl-CoA [67]; lipin 1 (LPIN1), which acts as a nuclear transcription coactivator regulating the expression of genes related to lipid metabolism [68]; and pantothenate kinase 1 (PANK1), which participates in CoA synthesis, important for β-oxidation [69, 70]. Accordingly, wild-type p53 increases fatty acid oxidation and oxidative phosphorylation, inhibiting Warburg effect.

### p53 and ROS regulation

Tumor cells have higher levels of ROS compared to normal cells and this may be useful to predict overall survival [71–75]. However, ROS induction has to be tightly controlled, because intermediate levels of oxidants can induce DNA damage, leading to mutation and promoting tumorigenesis. On the other hand, elevated levels of oxidants contribute to extensive DNA damage, mitochondrial membrane permeabilization, activation of apoptotic signaling and induction of cell death [76–78]. Cell metabolism is essential to control oxidants levels. In cancer cells, ROS modulate key metabolic enzymes, like pyruvate kinase M2 (PKM2) mentioned above [79, 80], inhibiting it through oxidation of Cys358, causing an increase in the availability of G6P and redirecting it to pentose phosphate pathway. This leads to formation of macromolecules and NADPH, which is required to generate GSH for ROS detoxification, controlling ROS levels in a cyclic mechanism [81].

There is an interplay between ROS and p53. The DNA damage induced by ROS can activate p53, which can both inhibit and promote oxidant production [82]. In tumor cells it was shown that low levels of p53 up-regulate several anti-oxidant genes, while down-regulation of p53 increases intracellular ROS and genome instability [83]. In normal conditions p53 protects cells from oxidative stress directly by inducing anti-oxidant genes, like glutathione peroxidase (GPX1), aldehyde dehydrogenase 4 (ALDH4) and Mn superoxide dismutase (Mn-SOD) [84–86] or indirectly by inducing TIGAR and GLS2. By favoring PPP, TIGAR also increases NADPH and GSH production and ROS scavenging [45]. GLS2 regulates antioxidant defense function in cells by increasing reduced glutathione (GSH) levels and decreasing ROS levels [60].

At the same time, p53 has been shown to activate the expression of genes that induce ROS and cell death. Among these a group of distinct genes were named p53 Induced Genes (PIGs) [87]. One of these genes, PIG3, is closely related to an NADPH-quinone oxidoreductase [87], it induces ROS [88] and decreases mitochondrial membrane potential [89]. Even though its induction is linked to apoptosis [90, 91], it requires other pro-apoptotic genes to induce apoptosis and it has also been proposed that PIG 3 may play a role in cancer cell survival, inducing sub-lethal levels of ROS [92], actively participating in the PI3K/AKT/PTEN pathway in PTC (papillary thyroid carcinoma). Silencing of PIG3 increased expression of PTEN and reduced PI3K and phosphorylated AKT, it was suggested that PIG3 induces ROS at intermediate levels, which induces phosphorylation of AKT and activation of mTOR [93]. We have observed that PIG3 is induced by p53 in prostate cancer, but its expression is not a key factor for induction of apoptosis [94].

As already discussed, POX, which participates in proline catabolism, is another p53-regulated protein. POX is also considered another member of the PIGs genes and may be called PIG6. POX catalyzes the conversion of proline into Delta-1-pyrroline-5-carboxylate (P5C) after p53 activation in colon and bladder cancer cell lines [95, 96]. PIG 6 also induces ROS, which mobilizes calcium and Calcineurin, causing apoptosis [97], it induces mitochondrial superoxide radicals and induce intrinsic pathway apoptosis, but also induces TRAIL, DR5 and caspase 8 cleavage by the extrinsic pathway [98]. Apoptosis mediated by POX can be inhibited by Mn-SOD induced by p53 [98, 99]. On an opposite effect it has also been observed that under hypoxia POX induces protective autophagy dependent on ROS, allowing tumor cell survival [100].

Another transcription factor involved in ROS inhibition is NRF2 (NF-E2 related factor 2). After oxidative stress this protein is stabilized and translocated to the nucleus, and induces the expression of antioxidant genes [101–104]. p53 modulates NRF2 activity, it can inhibit the expression of NRF2 through inhibition of Sp1 binding to the promoter region of NRF2 gene [105], inhibiting the activation of NRF2 antioxidants target genes, like x-CT, NQ01 and GST-α1 genes [106]. However, p53 has also been described to increase NRF2 activity indirectly through p21 induction. p21 directly interacts with NRF2 and prevents its degradation by inhibiting its ubiquitination [107]. Another review suggested that there are two phases of modulation of NRF2 by p53, in a first moment low levels of p53 induces p21 and therefore NRF2, in a second moment after severe DNA damage and high p53 expression inhibits the survival response mediated by NRF2 and induces cell death [108].

**Figure 3:**
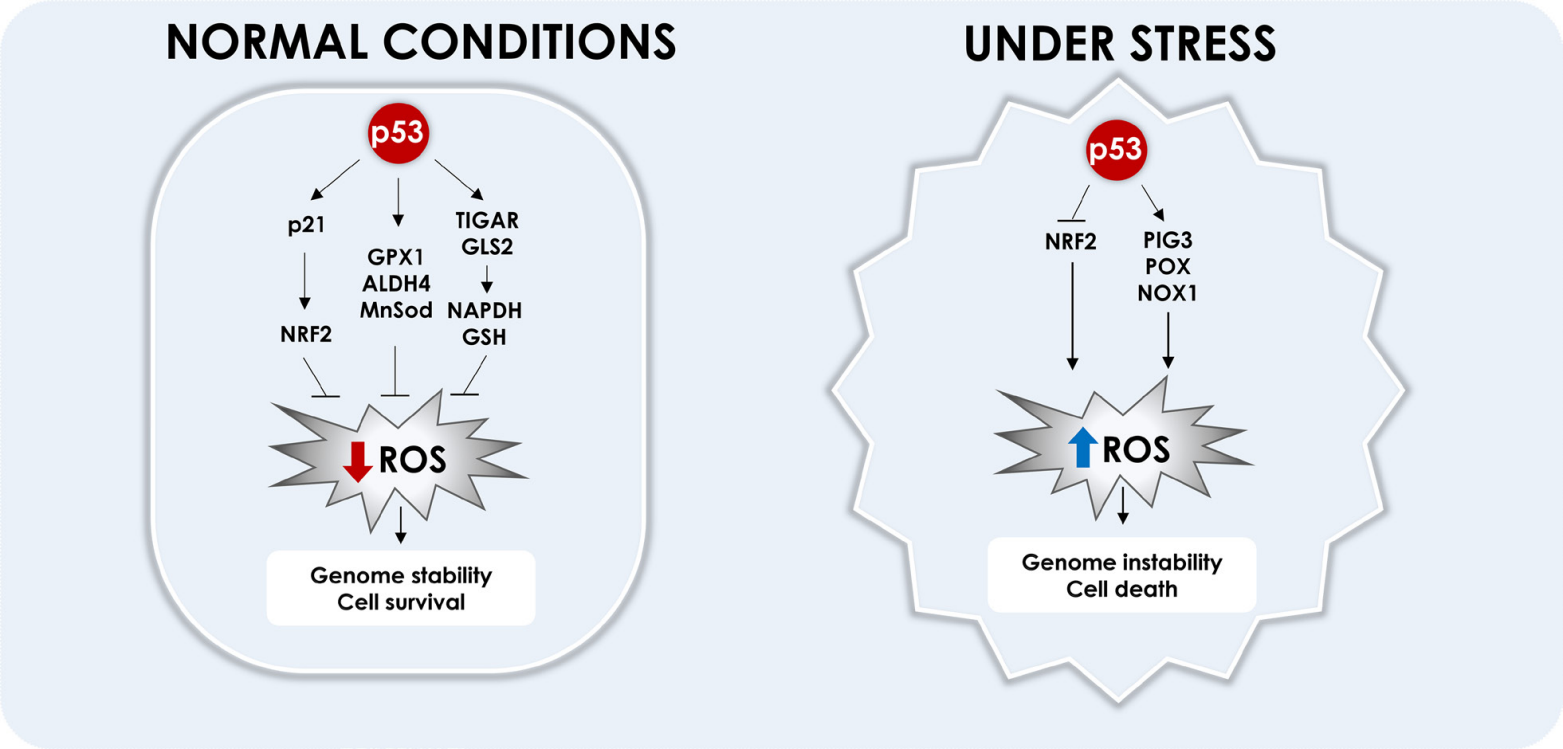
Modulation of ROS by p53 In normal cells, p53 was shown to reduce ROS levels, preventing DNA damage and promoting cell survival. p53 induces directly the anti-oxidant genes: GPX1, ALDH4 and Mn-SOD. p53 also induces TIGAR and GLS2, that redirects the cell metabolism to PPP, which increases NADPH and GSH production and ROS scavenging. p53 also induces p21 that prevents NRF2 degradation, which reduces ROS levels. In cancer cells or during stressful situations, p53 inhibits NRF2 expression and induces pro-oxidant genes: PIG3, POX and NOX1, which reduces mitochondrial membrane potential, induces DNA damage and apoptosis.

Several drugs that induce apoptosis of cancer cells rely on ROS induction [109, 110]. Indeed, an essential component of p53's mechanism of action is the induction of oxidants [82, 111–113]. ROS (peroxide and superoxide) induction mediated by p53 was shown to induce apoptosis independently of cytochrome-c release and can regulate mitochondrial membrane potential [114]. Induction of p53 by ROS has been shown to mediate necrotic cell death through PARP activation [115]. PIG3 and PIG6 are downstream targets of p53 that induce oxidants production; however, others and we have observed that expression of these genes is not essential to induction of apoptosis. We have observed that NADPH oxidase, especially NOX1, is induced by p53 and can induce apoptosis of prostate cancer cell lines [94]. Figure 3 shows how in normal conditions p53 activates genes involved in ROS detoxification and under stress p53 induces genes that increase ROS to toxic levels that induce cell death.

### p53 and growth factors signaling

Growth factors are essential for cell homeostasis but their signaling is frequently deregulated during the tumorigenic process, and they are usually targets of tumor suppressors, such as p53 [1]. Activation of the signaling pathway of insulin-like growth factors (IGFs) is responsible for stimulating cell growth, proliferation and metabolism, sending pro-survival signals and contributing to increased glucose uptake [116, 117]. Insulin-like growth factors 1 and 2 are recognized by IGF1R, which in turn activates the MAPK and PI3K/AKT/mTOR signaling pathways (Figure 1). In addition, they have their activity regulated by co-stimulatory IGFBPs (1-6) proteins that have different effects on IGF signaling [117]. When activated, p53 is responsible for inhibiting the transcription and activation of a wide range of genes related to this pathway, including IGF1 and IGF1R [118–120]. p53 promotes as well the transcription of AMPK subunits, Tuberous Sclerosis 2 (TSC2), PTEN and IGFBP1 and 3, known negative regulators of IGF1/AKT/mTOR pathway and tumor suppressors [121–124]. mTOR down-regulation may lead to less activation of sterol regulatory element-binding proteins (SREBP), which can also be further degraded by GSK3, whose activation happens through inactivation of AKT by p53 [125–128]. Those two mechanisms simultaneously lead to reduced metabolism of fatty acids and cholesterol biosynthesis pathways [127].

Besides promoting expression of negative regulators of mTOR pathway, after genotoxic stress, DNA damage or in response to oxidative stress, p53 leads to AMPK phosphorylation and activation [129, 130]. Active AMPK phosphorylates TSC2, thus inhibiting mTOR pathway. Its activation is dependent of Sestrin 1 and 2, whose transcription is controlled by p53 [129, 131]. Sestrins 1 and 2 also participate on autophagy [132–135], by activating AMPK, which phosphorylates ULK1, an autophagy initiation kinase, and at the same time through mTOR inhibition, which is a negative regulator of ULK1 [136].

In a feedback mechanism, activated AMPK is also capable to phosphorylate p53 and induce G1 arrest when the cell is under glucose deprivation, allowing the cells to survive until growth conditions are restored [137]. Moreover, if conditions do not improve, AMPK persistent activation leads to p53 dependent senescence [137]. Another feedback mechanism linking energy status, cell growth and p53, involves p38αMAPK stimulation after DNA damage, and consequent mTOR/S6K1 activation [138]. In this case, activated S6K1 interacts with MDM2, blocking its inhibitory effect over p53. Working as a negative feedback loop, p53 can inhibit mTOR activation, S6K1 then releases MDM2 turning off p53 activity (Figure 1) [131, 138, 139].

Nevertheless, different levels of p53 activation can trigger different outcomes. High levels inhibit mTOR inducing reversible quiescence instead of senescence or cell death, while low levels are not able to inhibit mTOR, and with prolonged cell cycle arrest can lead to senescence, what is essential to understand the action of different chemotherapeutic agents as will be described below [140].

Not only PI3K/AKT/mTOR pathway affects classic chemotherapy, but also immunotherapy, having a significant impact on immune checkpoint blockade, that target CTLA-4 and PD-L1. PD-L1 expressed on the surface or tumor cells inhibits T-cells proliferation and effector function after its recognition by PD-1 [141]. PDL1 expression is correlated with tumor aggressiveness for different types of tumor [142, 143]. Presence of PD-1 in tumor infiltrating lymphocytes is associated with poor survival [144]. A recent review indicated that PI3K/AKT/mTOR pathway inhibitors reduce expression of checkpoint ligands, and is also important for T-regs immunosuppressive function, suggesting that combination with anti-PD-L1 and inhibitors of PI3K/AKT/mTOR could favor both therapies [145]. Another important aspect is that PD-L1 expression is correlated with p53 status [146], probably because p53 can inhibit PD-L1 through miR-34a [147]. Therefore, p53 and PI3K/AKT/mTOR pathways affect immune checkpoint blockade therapies.

### Role of p53 in senescence and autophagy

Senescence is characterized by irreversible loss of the ability to proliferate in culture [148], and is more prone to happen in pre-malignant tumors. The senescent cells can be cleared by immune cells and have a more efficient tumor regression [149]. Interestingly senescent cells show reduced p53 transcriptional activity [150]. The first step for a cell to undergo senescence is related to p53 ability to promote cell cycle arrest through induction of p21, a cyclin-dependent kinase inhibitor that stops cell cycle at G1 phase [151]. However, cell cycle arrest is not enough to determine if a cell is senescent, as quiescent and differentiated cells also present the same characteristic [152]. Other senescence markers are senescence-associated beta-galactosidase and senescence-associated heterochromatic foci (SAHF); moreover p53 targets p21, promyelocytic leukemia protein (PML), plasminogen activator inhibitor (PAI-1) and deleted In Esophageal Cancer 1 (DEC1) [153].

Macroautophagy or simply autophagy is a process that controls degradation of proteins and organelles, necessary to recycle proteins and cell components and also represents an adaptive response to keep homeostasis after exposure to stressful conditions, it is essential for cell survival and at the same time its prolonged activation can also lead to cell death [154]. p53 can lead to autophagy as a protection against adverse growth conditions, keeping cells on a quiescent state until conditions improve. Inhibition of this protective autophagy redirects cells from quiescence to senescence on a mTOR dependent mechanism [155, 156].

Senescence and autophagy can be modulated by mTOR and p53. mTOR induces senescence and inhibits autophagy, while p53 was shown to induce autophagy, which inhibits senescence [156]. AKT was also shown to induce stabilization of p53 during nutrient starvation and silencing of p53 and AKT increased autophagy, indicating that the AKT-p53 axis may also play an opposite role in cell survival during nutrient starvation [157]. On Nutlin-3-resistant tumor cells, p53 activation leads to a protective autophagy and glycolysis stimulation, while on apoptosis sensitive cells, p53 activation decreases glycolysis, raises superoxide levels and inhibits autophagy. Using a combination of Nutlin-3 with glycolysis inhibitors, treatment of resistant tumor cells has the same effect presented on sensitive cells, with inhibition of autophagy and apoptosis triggering, indicating that metabolism plays an essential role in cell fate [158].

The switch from autophagy to apoptosis can be controlled by p53, regulating the expression pattern of autophagy related genes, ULK and ATG family [43], and apoptosis related ones, Bcl2, PUMA, Bax and others [159] depending on its activation signal. When it's phosphorylated on Ser15, p53 dissociates from MDM2, inhibits Beclin1 and LC3 and activates apoptosis and inhibits autophagy [160]. In addition, when phosphorylation occurs on S392, p53 inhibits ULK1 directly, switching autophagy to apoptosis [161].

Autophagy usually has a protective effect against critical environmental conditions, allowing cells to survive until conditions improve. Nevertheless, cells can survive after losing the ability to activate autophagy and when it happens together with p53 inactivation this might trigger adenocarcinoma development, with changes in the metabolism focusing on anabolic pathways [162]. In fact, the relationship between autophagy and p53 determines cell fate, limiting tumor development when autophagy is blocked and p53 is functional, and promoting progression from benign lesions to invasive tumor when there is a lack of p53 combined with autophagy genes silencing [163].

In the end, if autophagy will prevent cells from dying or not depends on p53 status [164], and which will be the cell fate, autophagy, senescence, apoptosis, quiescence, depends of p53 combined with microenvironment conditions and other pathways alterations presented in the cell [165–169].

### Modulation of p53 activity

p53 transcriptional activity can be modulated by its interaction with other transcription factors. One important interaction partner is the Peroxisome proliferator-activated receptor γ Coactivator 1-α (PGC-1α) protein. Interaction of PGC1α with p53 directs the expression of genes involved in cell cycle arrest and metabolism [170]. PGC1α positively regulates respiration, gluconeogenesis, mitochondrial biogenesis, metabolic processes and ROS inhibition [171–173]. Induction of PGC1α by starvation caused activation of the following p53 target genes: p21, GADD45α, TIGAR, SCO2 and Sestrin2 at early time points, later, if starvation continued, pro-apoptotic genes like BAX, PUMA and NOXA were activated. Silencing of PGC1α abrogated the induction of the pro-arrest and metabolic genes, while the pro-apoptotic genes were not altered [170]. There´s a feedback mechanism in these situations, p53 binds to and induces PPARGC1a promoter and induces expression of PGC1α. Depletion of the antioxidant GSH induced the p53-PGC1α-NFR2 axis [174]. It's interesting, however, that p53 have also been shown to inhibit PGC1α, and induce oxidative stress of cardiomyocytes [175].

### Mutant p53

Since the early studies of p53, It's evident that its mutant forms may be just as important as the wild-type for cancer. Mutant forms of p53 have different expression profiles and may activate tumorigenic genes, showing an oncogenic “gain of function”, exhibiting a dominant-negative effect over the wild-type protein [176]. A recent review points out that there's a “rainbow of mutants” of p53 and each one has a particular degree of gain of function and pathological consequence, implicating that knowledge of the specific mutations may have an impact on the therapeutic approach decision [177]. These mutant forms of p53 also regulate tumor cell metabolism and may modulate drug sensitivity.

Warburg effect and shift of cell metabolism to glycolysis are directly affected by p53. Expression of hexokinase 2 (HK2), the first enzyme of the glycolytic pathway, is induced by mutant p53, increasing the conversion of glucose to glucose-6-phosphate (G6P), which may diverts to glycolysis or PPP [178]. Cells expressing the mutant p53 increased expression and phosphorylation of PKM2 mediated by mTOR, while wild-type p53 had no positive influence in the expression levels of PKM2 and knockdown of wild-type p53 increased PKM2 phosphorylation [179]. Mutant p53 also increases PKM2 and promotes hepatocarcinogenesis [180]. The p53 target gene TIGAR that is upregulated by wild-type p53, is downregulated by different p53 mutants, including mutations in the DNA binding domain [181, 182] or mutations in the C-terminal, comprising the tetramerization and regulatory domains [44]. This differential expression of TIGAR in wild-type and mutant p53 has an impact in cancer treatment. The high expression levels of TIGAR mediated by wild-type p53 reduced the dependence on the glycolytic pathway and FDG efflux into the tumor cell. Cells expressing mutant p53 showed an increased dependence on the glycolytic pathway and sensitivity to the action of FX11, an inhibitor of the enzyme Lactate Dehydrogenase, which is responsible for the conversion of pyruvate to lactate and in the process regenerates NAD^+^ and divert pyruvate from conversion to acetyl-CoA that is important for oxidative phosphorylation [183]. Expression of mutant p53 increased glucose uptake, glycolytic rate and lactate production. These mutant forms promote GLUT1 and GLUT 4 translocation through RhoA/Rock activation. Inhibition of the RhoA/Rock/Glut1 axis abolished Warburg effect mediated by mutant p53 [184].

Garritano *et al.* compiled the results from the literature of wild-type and mutant p53 expression [185]. In their work, it was observed that POX, GLS2, SESN1, GAMT, NOTCH1, NOTCH3 and PLA2G2A have altered expression in cells expressing mutant p53. Pointing out the importance of GLS2 and POX to modulate metabolism to increase glycolysis and directing to Warburg effect. Tepper *et al*. [186] identified that PGM presents increased expression mediated by mutant p53. Several metabolic intermediates are tightly regulated by p53. Enzymes regulated by wild-type p53 including GAMT, GLS2 and POX are negatively regulated by mutant p53 [181, 182, 185]. In the high-grade serous ovarian cancer (HGS-OvCa) the p53 gene is mutated in almost all cases, reaching 96% [187]. It has been proposed that in HGS-OvCa the mutant p53 promotes sterol regulatory element binding proteins (SREBPs) and inhibits GAMT, promoting lipid anabolism and fatty acid oxidation [188]. As a net result there is a promotion of lipid anabolism that accelerates tumor growth and progression [188].

In normal cells, p53 exerts an important role in ROS detoxification, maintaining low levels of oxidants; while tumor cells exhibit increased levels of ROS. Cells expressing mutant p53 show repression of several important genes involved in ROS inhibition. Cells expressing mutant p53 showed a reduced expression of ALDH4A1 [182]. p53R273H reduced the NRF2 activity resulting in defect in ROS detoxification and cell survival [189], while mutants p53H179Y, p53L194R, p53S240R, p53R249S, p53A276D, and p53E286Q had no influence in the levels of NRF2 [105]. SESN1 and SESN2 expression was reduced by mutant p53 [44, 181, 185]. Some p53 mutants were not able to induce POX and activate the calcineurin pathway and apoptosis [97]. Downregulation of different genes involved in ROS detoxification may me important to maintain the intermediate levels of ROS that favors tumor cell growth.

Expression of the PGC1α transcription factor was reduced in cells expressing p53 missense mutations or with loss of p53 in a comparison of 28 lung adenocarcinoma cell lines with different p53 status [190]. However, one particular mutation p53P72R showed increased PGC1α function and increased metastatic capability [191]. Even though this mutation didn't show evidence of increased glucose regulation compared to wild-type p53 [192].

The mTOR pathway is also modulated by p53. One of the main mechanisms involved in this regulation is through phosphorylation of AMPK. Inhibition of AMPK phosphorylation results in stimulation of the mTOR pathway. Expression of mutant p53 inhibited BECN1, DRAM1, ATG12, SESN1/2, repressed AMPK phosphorylation, increased phosphorylation of p70S6K and reduced autophagy [193, 194]. AMPK phosphorylation can also be induced by Sestrins [129, 195, 196], which have been observed to be increased by mutant p53 knockdown [194]. mTOR activity can also be modulated by EGFR, it has also been shown that p53R280K has an increased EGFR signaling [197]. Using an inhibitor of mTOR (Everolimus), the cells expressing mutant p53 were more sensitive to the drug, evidencing a stronger dependence in mTOR pathway [194]. Phosphorylation of AKT is induced by the mutant p53R273H, but not p53R175H [198]. In general, mTOR and S6Ks, known mediators of mTOR signaling, have been related to cancer progression in several cancer models [199–201].

Mutant p53 represses autophagy and is dependent on its cellular localization. Some mutant p53 have a preferential nuclear localization and fail to repress autophagy, while mutants that show a more cytoplasmic localization are able to repress autophagy. Mutants that have both cytoplasmic and nuclear localization are also able to inhibit autophagy, indicating that repression of autophagy occurs in the cytoplasm [202]. Interestingly while wild-type p53 is degraded through proteasome pathway, it was shown that mutant p53 (more than 20 different p53 forms have been tested) is deacetylated and degraded through lysosomal-dependent pathway in a chaperone-mediated autophagy (CMA) or through macroautophagy [203–205]. Collectively these data indicate that mutant p53 inhibits autophagy, which is responsible for the degradation of mutant p53, therefore prolonging its half-time.

**Figure 4:**
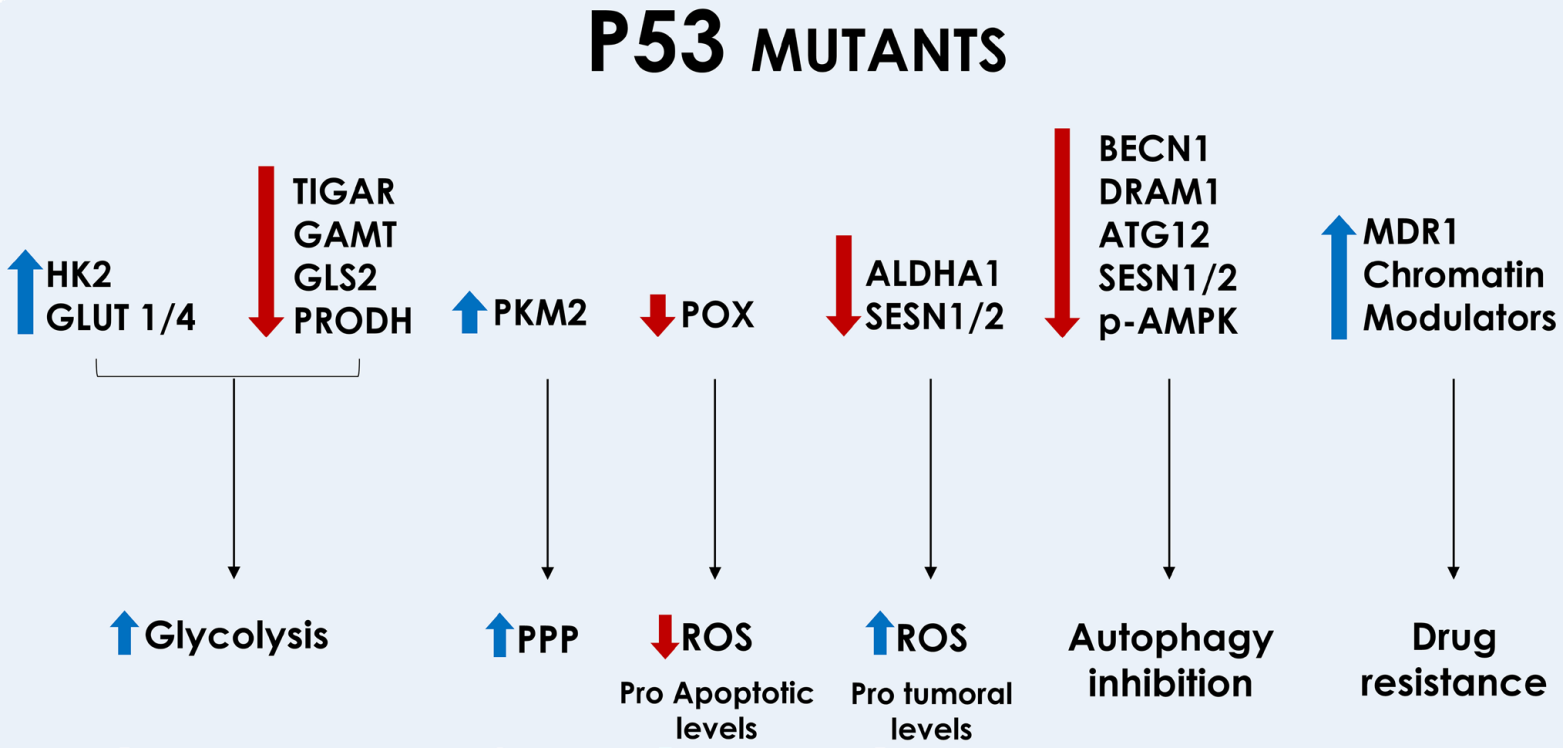
Modulation of metabolic genes by mutant forms of p53 Mutant p53 increases glycolysis through inhibition of TIGAR, GAMT, GLS2 and POX; and through induction of HK2 and GLUT1/4 translocation. Redirection of the PPP can be achieved through induction of PKM2. Some mutants of p53 reduce POX and ROS induction and ROS levels can be increased through inhibition of ALDHA1 and SESN 1/2. Autophagy is inhibited through downregulation of BECN1, DRAM1, ATG12, SESN1/2, and reduction of AMPK phosphorylation. Increased drug resistance is mediated by induction of MDR1 and chromatin modulators.

Mutant p53 has also been shown to be important to increase drug resistance through different mechanisms. Mutant p53 induces activation of the Multi-drug resistance (MDR1) promoter. The MDR1 is a drug efflux pump that participates in the removal of drugs from the cell [206–208]. Patients with colorectal carcinoma showed a positive correlation among mutant p53, MDR1 (P-gp, ABCB1) and GST-pi expression [209]. Oxidative phosphorylation was shown to inhibit expression of the ABC transporters in cells expressing wild-type p53 and to increase expression of these transporters in cells expressing mutant p53 [210]. Increased drug resistance may also be modulated by mutant p53 through modulation of the expression of pro and anti-apoptotic genes like Fas and Bcl-XL [211]. Another important class of genes induced by mutant p53 is chromatin modulators. Post translational modification of histones are essential to regulate gene expression; methylation or acetylation of H3K4 are involved in gene activation, while methylation or acetylation of H3K9 and H3K27 are involved in gene repression [212]. The MDR1 is one of the genes activated by H3K4 methylation and acetylation [213]. Among the genes repressed by methylation of H3K9me3 and H3K27me3 are several genes involved in induction of apoptosis (FAS, Bcl2 and BAX). Silencing of KDM5D (JARID1D) (H3K4me3 demethylation agent) increases drug resistance [214]. As a net result, there is an increased chemoresistance [215–217]. The p53 GOF mutants induce expression of genes involved in histone methylation (H3K4me3 and H3K4me1) by MLL1 (kmt2a) and MLL2 (kmt2d) and histone acetylation (H3K9ac) by MOZ (kat6a) [218]. Mutant p53 induces PKM2, which interacted with H3 and caused H3K9me1 and H3K9Ac (Figure 4) [180]. Indicating that mutant p53 activates chromatin regulatory genes that repress important pro-apoptotic genes and induce drug efflux pumps. Figure 4 shows how mutant p53 induces or inhibits different genes that ultimately results in increased glycolysis, PPP, inhibition of autophagy, drug resistance and reduction of pro-apoptotic ROS and increase in pro-tumoral levels of ROS. Supplementary Table 1 lists the capacity of mutant p53 forms to induce/inhibit genes involved in metabolism, ROS and growth control, drug sensitivity, autophagy and cellular localization.

## THERAPY TARGETING METABOLISM

### Glucose metabolism

**Figure 5:**
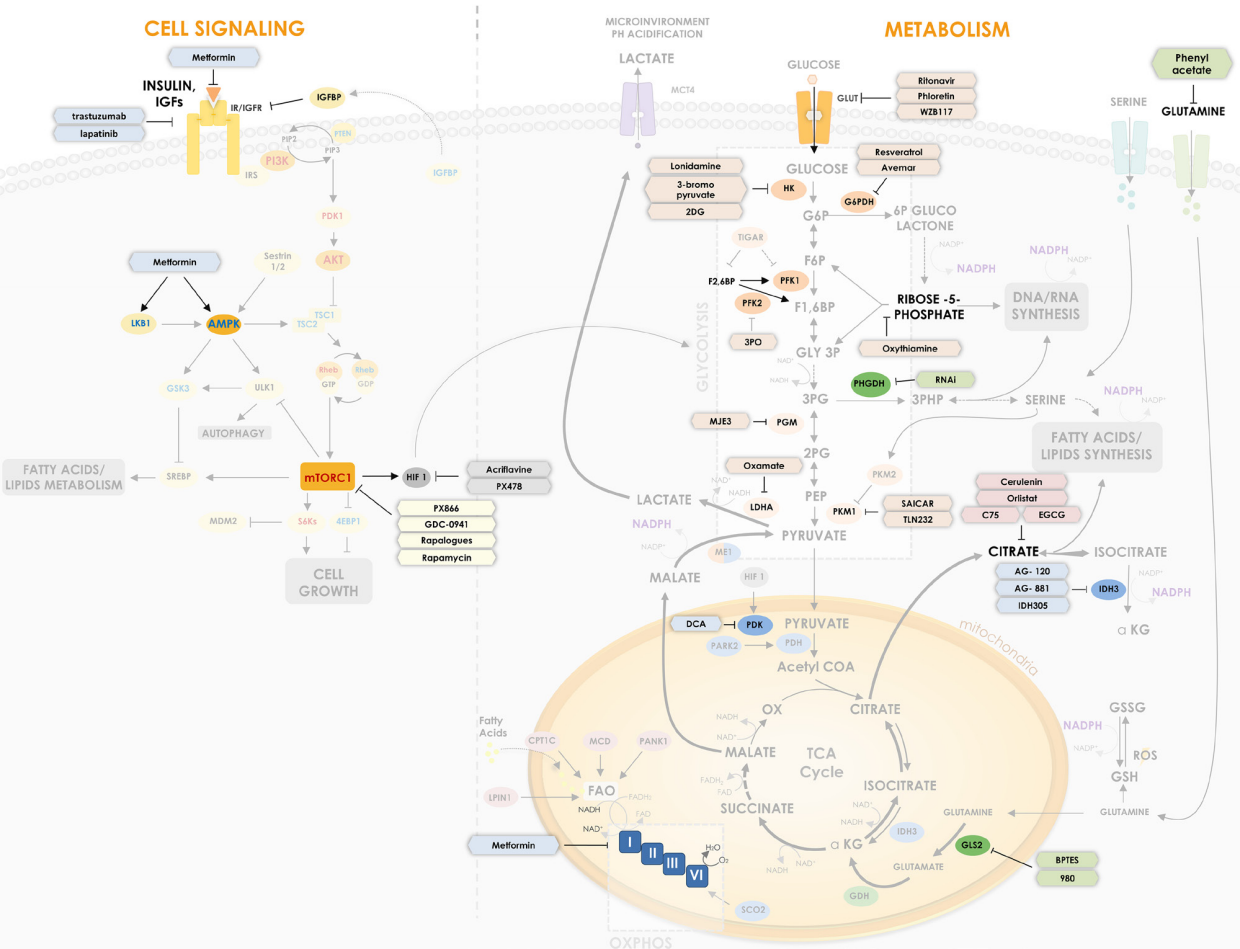
Metabolism based cancer therapies targets Different therapies are being developed to regulate crucial metabolic targets. Some therapies are already validated for other diseases, such as metformin and DCA. The Figure shows molecular targets that are currently under therapeutic investigation in cancer. Drugs, their targets and relation with p53 are summarized in Table I.

The molecular targets related to alterations in cancer metabolism that are currently under therapeutic investigation are summarized in Table 1 and Figure 5 correlates the drugs mentioned bellow with their targets in the metabolic pathways. As already mentioned, members of glucose transporters of the GLUT family are overexpressed in several types of cancers, suggesting that these transporters could be possible therapeutic targets [219]. Ritonavir, an HIV protease inhibitor that displays off-target inhibitory effects on GLUT4, can reduce the viability of myeloma cells and increase the sensitivity to the chemotherapeutic doxorubicin [220]. Ritonavir administration is associated with perturbation in the proteasomal activity and a small effect in p53 accumulation in several types of cancer [221]. It has also been found that Ritonavir inhibits AKT and induces senescence and oxidative stress of PBMCs [222, 223]. Phloretin is a naturally occurring dihydrochalcone that inhibits GLUT1 and, under hypoxia conditions, enhances daunorubicin anticancer effects [224]; it also induces cell cycle arrest in a p53 dependent manner [225]. WZB117 is another inhibitor of GLUT1 that leads to a lowered rate of glycolysis and cellular growth, showing synergistic anticancer effects when combined with cisplatin or paclitaxel, both drugs are known p53 inducers [226].

**Table 1:** Drugs acting over metabolism and the existence or not of a relation with p53

Glucose metabolism					
**Compound**	**Target**	**Effects**	**Stage of development**	**Relation with p53**	**References**
Ritonavir	GLUT4	Inhibits GLUT4	Clinical trial	Yes	[220]
Phloretin	GLUT1	Inhibits of GLUT1; enhances daunorubicin's anticancer effects	Preclinical	Yes	[224]
WZB117		Inhibits GLUT1	Preclinical	Yes	[226]
2DG	Hexokinase	Inhibits glucose metabolism	Preclinical	Yes	[247–249]
3-bromopyruvate		Inhibits glucose metabolism	Preclinical	Yes	[250]
Lonidamine		Inhibits glucose metabolism; enhances doxorubicin anticancer effects	Clinical trial	Yes	[254–255]
siRNA	PFK2	Inhibits glucose metabolism	Preclinical	Yes	[256]
Phosphonomethyl analogue	Phosphoglycerate mutase	Inhibits glucose metabolism	Preclinical	Yes	[48–49]
shRNA	Pyruvate kinase	Improves the therapeutic efficacy of cisplatin and docetaxel	Preclinical	No	[261]
TLN232		Inhibits glucose metabolism	Clinical trial	No	[262]
SAICAR		Indirectly mediates antitumor effects	Preclinical	No	[263–264]
Oxamate	LDHA	Inhibits LDHA	Preclinical	No	[267]
shRNA	MCT4	Blocks the adaptive response to antiangiogenic drugs	Preclinical	No	[268–269]
Phloretin		Inhibits MCT4	Preclinical	No	[270]
**Lipid metabolism**					
Cerulenin	FASN	Accumulates malonyl-CoA and induces ER stress	Preclinical	Yes	[275, 277–278, 280]
C75		Accumulates malonyl-CoA and induces ER stress	Preclinical	Yes	[276–277, 279–280]
Orlistat		Irreversibly inhibits the FASN activity, blocking FA synthesis	Approved for obesity	Yes	[282–285]
EGCG		Inhibits 50% of FASN activity, leading to apoptosis	Clinical trial	Yes	[281–285]
SB-204990	ACLY	Inhibits cholesterol and FA synthesis	Preclinical	Yes	[291]
ND-646	ACC	Inhibits ACC enzymes (ACC1 and ACC2), suppressing FA synthesis	Preclinical	Yes	[292]
HC-3	CK	Inhibits phosphatidylcholine synthesis	Preclinical	No	[293–294]
CK37		Reduces the concentration of phosphocholine in transformed cells	Preclinical	No	[293]
**Amino acid metabolism**					
Phenylacetate	Glutamine	Reduces the biodisponibility of glutamine in the blood.	Preclinical	Yes	[297, 300]
BPTES	GLS	Inhibits GLS	Preclinical	No	[301]
968		Inhibitor of Rho GTPase-dependent cellular transformation, targets GLS	Preclinical	No	[303]
Arginine deiminase	Arginine	Converts arginine to citrulline and ammonia	Clinical trial	Yes	[307]
Asparaginase	Asparagine	Converts asparagines to aspartate and ammonia	Approved	Yes	[311–314]
RNAi	PHGDH	Inhibits PHDGH	Preclinical	No	[25–26]
**Krebs cycle and oxidative phosphorylation**					
DCA	PDK1	Metabolic shift from cytoplasm-based glycolysis to mitochondria-based glucose oxidation	Clinical Trial	Yes	[316–322]
Metformin	Mitochondrial complex I, AMPK	Activates AMPK since decreases mitochondrial respiration chain activity and ATP production	Approved for diabetes and polycystic ovaries syndrome	Yes	[330–349]
HMS-101	IDH	Specifically inhibits Isocitrate Dehydrogenase (IDH)	Preclinical	No	[323]
AG-120		Specifically inhibits Isocitrate Dehydrogenase (IDH)	Clinical trial	No	[324–325]
AG-881		Specifically inhibits Isocitrate Dehydrogenase (IDH)	Clinical trial	No	[324–325]
IDH305		Specifically inhibits Isocitrate Dehydrogenase (IDH)	Clinical trial	No	[324–325]
**Cell growth signaling**					
Lapatinib	EGFR	Inhibits EGFR	Preclinical	Yes	[350–352]
Trastuzumab			Preclinical	Yes	[350, 352]
Rapamycin	mTOR	Inhibits mTORC1 and enhances the antitumor effects of cisplatin	Approved as immunosuppressant	Yes	[353–355]
Rapalogues		Inhibits mTOR, stimulating autophagy	Approved as immunosuppressants. Temsirolimus and everolimus were approved for renal cell carcinoma, neuroendocrine tumors of pancreas and subependymal giant cell astrocytoma	Yes	[356]
Acriflavine	HIF-1	Inhibits HIF-1	Preclinical	Yes	[360–363]
PX478			Preclinical	No	[360–361, 363]
GDC-0941	PI3K	Inhibits PI3K/AKT/mTOR axis	Clinical trial	No	[270] [270]
PX866		Clinical trial	No		

G6PDH, the rate-limiting enzyme of PPP, allows the production of important molecules like NADPH and R5P, and is involved in the maintenance of GSH cellular levels. Breast cancer cells show a significant increase in oxidative PPP flux [227]. Over-expression of G6PDH leads to a significant resistance to apoptosis [228]. There are currently no inhibitors of PPP in clinical trials, even though it may be an attractive target. Some compounds like Resveratrol and Avemarare are known inhibitors of PPP enzymes like G6PDH [229, 230] and showed some positive results in colon cancer, leukemia and polycystic ovary syndrome cells by induction of apoptosis and cell cycle arrest [231, 232]. In 1999, an article described for the first time that Resveratrol increases endogenous levels of p53 in a cancer model [233]. The antiproliferative and pro-apoptotic role of Resveratrol has been demonstrated in several cancer types to be mediated by p53, including liver cancer, osteosarcoma and thyroid cancer [234–236]. In prostate cancer cells, Resveratrol induces cell cycle arrest and apoptosis when combined to docetaxel through p53 [237]. Finally, a study has demonstrated that wild-type and functional p53 is important to increase the anticancer effects of Resveratrol in cancer cells [238].

Transketolase-like protein 1 (TKTL1), an enzyme of the non-oxidative branch of the PPP, has been found to be upregulated in several tumor types [239], and is involved in tumor cell proliferation [240], sensitivity to ROS [241], and metabolic alteration mediated by TIGAR [242]. Oxythiamine, a thiamine antagonist, is able to bind TKTL1 and hinder tumor growth through inhibition of PPP and induction of cell cycle arrest [243, 244]. Thiamine antagonists are also able to trigger upregulation of p53 expression, phosphorylation and activation [245].

HK2 plays an important role both in glycolysis and apoptosis and its increased activity has been reported in several types of cancers, showing a direct relation between growth rates and HK2 activity [246]. 2-deoxy-d-glucose (2DG), an inhibitor of glucose metabolism, is a glucose analog that is phosphorylated by HK2, inhibiting it by competition and leading to growth arrest and/or apoptosis [247]. In addition, 2DG can potentiate the cytotoxic effects of chemotherapy, since combination of metformin and 2DG restored p53 function, inhibiting the overexpression of MDM2 and MDM4 and leading to cell growth arrest and apoptosis in MCF-7 cells resistant to doxorubicin [248, 249]. 3-bromopyruvate (3BP), another glycolysis and TCA inhibitor that targets HK2, leads to a depletion of the cellular ATP reserves, which was shown as a key determinant of chemoresistance in certain types of cancer [250]. In fact, multi-drug resistant cells have high expression of P-gp (P-glycoprotein 1), which requires ATP for its activity and therefore are more sensitive to ATP depletion [251, 252]. This inhibition of glycolysis not only enhances the cytotoxic effects of certain chemotherapeutics like daunorubicin and doxorubicin, but also markedly suppresses tumor growth when used with doxorubicin by limiting ABC transporters, like P-gp [253]. Lonidamine, a derivative of indole, is also an HK2 inhibitor. Results of clinical trials have shown that lonidamine combined with doxorubicin have good therapeutic efficiency for the treatment of certain types of cancer like breast, prostate, melanoma, brain and ovarian cancer [254]. 3BP and lonidamine presented diverse effects regarding p53 in glioblastoma cells. The first led to early cell death marked by p53 dephosphorylation, while the latter led to p53-dependent apoptosis favoring p53 translocation to mitochondria [255].

p53 promotes nucleotide biosynthesis through PPP in response to DNA damage by repressing the expression of FB3 isoform of PFK2 (PFKFB3), which is expressed in several cancers and is required for anchorage-independent growth of Ras-driven tumors [256]. Small-molecule inhibitors of PFKFB3, like 3-(3-pyridinyl)-1-(4-pyridinyl)-2-propen-1-one (3PO), have been reported to present a cytostatic effect on cancer cells [257].

As already discussed, PGM is upregulated by mutant forms of p53 [186] and regulates a unique step in glycolysis. Most of the glycolytic intermediates that are used as precursors for biosynthesis are upstream of this step. In several types of cancers, like hepatocellular carcinoma and colorectal cancer, its activity is increased [258]. The compound called MJE3 is a potent competitive inhibitor of PGMs that studies suggest to be a good candidate for chemotherapy [259, 260].

The pyruvate kinase isoform M2 (PKM2) is predominantly expressed in cancer cells as already mentioned. Several studies have shown a negative correlation between PKM2 expression and drug resistance, like cisplatin [261], suggesting that PKM2 is a potential target for adjuvant cancer therapy. TLN232 is an inhibitor of pyruvate kinase and has been evaluated as a stand-alone therapeutic intervention in patients with melanoma or metastatic renal cell carcinoma (ClinicalTrials.gov identifiers: NCT00735332; NCT00422786) [262]. Succinyl aminoimidazole carboxamide ribose-5’phosphate (SAICAR), an intermediate of the *de novo* purine nucleotide synthesis pathway, and the amino acid serine are known activators of PKM2, limiting the diversion of glycolytic intermediates towards the PPP and suggesting anticancer effects of serine starvation [263, 264].

Lactate dehydrogenase A (LDHA), the enzyme that converts pyruvate to lactate, is overexpressed in cancer, since the shift to oxidation of glucose to lactate is a well-known characteristic of the Warburg effect. The transcription factors, hypoxia-induced factor 1 (HIF-1) and Myc, known oncogenes, induce expression of LDHA [265]. The knockdown of human papillomavirus oncoprotein E6 in HeLa cells increased expression of p53 and decreased levels of miR-34a, which targets LDHA, thus reducing the Warburg effect [266]. Oxamate is a pyruvate analog that inhibits the conversion of pyruvate to lactate and has been reported to reverse the resistance associated to paclitaxel treatment associated with LDHA (Figure 5) [267].

Monocarboxylate transporter 4 (MCT4) is a membrane protein that exports lactate, allowing the preservation of cell pH homeostasis, and creating an acidic tumor extracellular environment [9]. Cells under hypoxia produce large amounts of lactate through LDHA, as mentioned before, and export them using MCT4. When oxygenated, cells may then absorb lactate from the extracellular medium using the MCT1 isoform, and convert it back to pyruvate for further oxidation [268]. Studies have shown that MCT4 knockdown could block the adaptive response to antiangiogenic drugs by disrupting the glycolytic flux [268, 269]. Phloretin, mentioned before, also has the capacity to inhibit MCT4, although mechanism insights and clinical trials are needed [270].

### Lipid metabolism

As already discussed, deregulation of the lipid metabolism is one of the most common alterations in cancer, since fatty acids are required by cancer cells to form membranes and signaling molecules. As shown in Figure 5, the citrate generated in the TCA cycle is exported from the mitochondria to the cytosol, where the enzyme ATP citrate lyase (ACLY) converts it to acetyl-CoA, which is subsequently converted to cholesterol or catalyzed by acetyl-CoA carboxylase (ACC) to form malonyl-CoA. Fatty acid synthase (FASN) is a key enzyme that acts in the conversion of malonyl-CoA to fatty acids. In normal adult tissues, FASN expression is very low, while in several types of cancer, such as prostate and breast cancer, it is significantly upregulated [271, 272]. In bone tumors, FASN expression is significantly increased in a p53 and MAPK-dependent manner [273]. High expression of FASN is associated with the development and survival of tumor cells, as well as a poor cancer prognosis [274]. These data suggest that FASN may be a therapeutic target for cancer.

FASN inhibitors, such as cerulenin, C75, orlistat and epigallocatechin gallate (EGCG), demonstrated anti-tumor activity, leading to toxic accumulation of the malonyl-CoA intermediate, reduction of membranes synthesis and phospholipid function. Cerulenin, isolated from *Cephalosporium caerulens*, contains an epoxy group that irreversibly binds to FASN leading to its inhibition [275]. C75, derived from cerulenin, also interacts with FASN promoting its inhibition [276]. Both cerulenin and C75 lead to apoptosis by promoting endoplasmic reticulum stress and malonyl-CoA accumulation [277]. However, the anticancer effects of cerulenin is limited by its chemical instability and lack of systemic activity [276]. In combination with cerulenin, the p53-p21 pathway activation by oxaliplatin occurred in a smaller concentration and induced caspase-3 cleavage [278]. Growth arrest induced by C75 is modulated by p38 MAPK but not by p53 in human hepatocellular carcinoma [279]. Finally, cancer cells exposed to cerulenin or C75 were sensitized by the loss of p53, indicating that these compounds may be clinically useful against malignancies carrying p53 alterations [280].

Orlistat is classified as an anti-obesity drug, although recent studies have shown its anticancer effects, since orlistat is able to irreversibly inhibit FASN [281]. Orlistat promotes apoptosis, reduces cell growth and proliferation, angiogenesis and metastasis and modulates the expression of several genes including p53, Bcl2, PUMA and Caspase-3 [282–284]. Despite these effects, orlistat has a poor bioavailability and non-specific side effects, which may be a limiting factor for its use as an anticancer drug. Combinations of orlistat with other drugs have been studied in order to optimize its anticancer effects [285]. EGCG derived from green tea, is also an inhibitor of FASN activity and has anti-tumor effects, inducing apoptosis in several cancer cell lines and reducing tumors size [286, 287]. EGCG is able to induce apoptosis in the presence of wild-type and mutant p53, indicating that a p53-independent pathway may contribute to EGCG-induced apoptosis in colon cancer cells [288]. A study confirmed that the combination of EGCG and an siRNA targeting p53 in triple-negative breast cancer cells leads to activation of pro-apoptotic genes and the inhibition of pro-survival genes [289].

High expression of ACLY in cancer is observed, suggesting its oncogenic role, and ACLY silencing in cancer cells induced p53 activation and facilitated DNA damage-induced cell death, suggesting it may be a therapeutic target [290]. SB-204990, an ACLY inhibitor, reduces proliferation and survival of cancer cells, *in vitro*, and tumor growth and cells differentiation, *in vivo* [291]. An allosteric inhibitor of ACC, ND-646, suppresses fatty acid synthesis and tumor growth of non-small cell lung cancer, even in p53 null cells, in preclinical models [292].

Choline kinase (CK) participates in the phosphatidylcholine synthesis, which is an important constituent of the cell membrane. CK is overexpressed in tumors and is considered a potential target for cancer treatment [293]. Hemicholinium-3 (HC-3) is an inhibitor of CK activity, that has an oxazinium ring that occupies the choline binding site and is described to have anti-proliferative effects [294]. Another CK inhibitor, CK37, is able to disrupt the actin cytoskeleton, promoting ultrastructural changes in the plasma membrane and inhibiting cell proliferation and tumor growth [293].

### Amino acids metabolism

Glutamine, as mentioned before, is highly consumed in cancer cells and may be oxidized to lactate. Its metabolism is an important source of GSH for redox control, citrate for fatty acids biosynthesis and TCA cycle intermediates for amino acids biosynthesis. Phenyl acetate is a drug that reduces the bioavailability of glutamine in the plasma, by condensing with the γ-amino group of glutamine and leading to its excretion in urine [295]. Phenylacetate inhibits the proliferation of tumor cells and promotes their differentiation in a phenotype that is usually associated with less aggressiveness [296]. A point to consider, however, is that glutamine depletion from the plasma may also increase cachexia conditions [297]. A Phenylacetate derivative called SCK6 inhibits proliferation of human lung cancer cells via G1 cell cycle arrest and apoptosis mediated by p53 and p21 [298]. In breast cancer and prostate cancer cells, however, phenylacetate increased p21 and p27Kip1 expression, respectively, without affecting the expression levels of p53 [299, 300].

GLS2, a key enzyme of the glutaminolysis process in rapidly growing cancer cells, is also an important therapeutic target. Bis-2-(5-phenylacetamido-1,2,4-thiadiazol-2-yl) ethyl sulfide (BPTES) is an allosteric inhibitor of GLS [301]. This compound preferentially inhibits the growth of cells with mutant isocitrate dehydrogenase IDH1 [302], but BPTES also decreases aerobic cell proliferation through induction of hypoxic cell death [267]. 968, a GLS inhibitor that is dependent on Rho GTPases, hinders the growth of human breast cancer and B lymphoma cells without affecting normal cells (Figure 5) [303].

Arginine is a non-essential amino acid in normal tissues, however, at least two types of cancer, hepatocellular carcinoma (HCC) and melanoma, require exogenous sources of arginine, leading to asparagine auxotrophy [304]. Those cancer cells do not express argininosuccinate synthetase 1 (ASS1), which is essential for endogenous arginine synthesis [305]. Therefore, depletion of arginine in the plasma of patients adversely affects the growth of HCC or melanoma tumors [306]. Clinical tests are exploring the anticancer effects of arginine deiminase, which converts arginine to citrulline and ammonia, resulting in the depletion of arginine and demonstrating anticancer effects [307]. Arginine deiminase upregulates p53 and p27Kip1 and downregulates cyclin D1, c-Myc and Bcl-xL in stomach adenocarcinoma cells [308]. In colon cancer cells, administration of arginine deiminase causes a p53-dependent cell cycle arrest by induction of microRNA-16 [309]. Finally, expression of arginine deiminase by a human telomerase reverse transcriptase promoter presented higher hepatoma targeting and oncolytic efficiency than expression of p53 by the same promoter *in vivo* [310].

Asparagine, like arginine, is a non-essential amino acid in humans, due to the presence of the asparagine synthetase (ASNS). In certain types of cancer, including leukemia, the activity of ASNS is decreased and cancer cells require asparagine uptake from the plasma to survive [304]. Asparaginase, a recombinant bacterial enzyme that converts asparagine to aspartate and ammonia, reducing the plasma levels of the first, has been approved by FDA for the treatment of childhood acute lymphoblastic leukemia [311]. The combination of 6-thioguanine, arabinoside and PEG-asparaginase is able to downregulate Bcl2 oncoprotein levels in both p53-null or p53-expressing leukemia cell lines [312]. In acute lymphoblastic leukemia cells, inhibition of autophagy enhances L-asparaginase-induced cytotoxicity and overcomes the acquired resistance to L-asparaginase, whereas a ROS-p53-positive feedback loop is an essential mechanism of this synergistic cytotoxicity [313]. Finally, the combination of asparaginase and the Nutlin RG7112, a known inhibitor of p53-MDM2 interaction that will be more discussed later, presented therapeutic enhancement against mixed-lineage leukemia-acute lymphoblastic leukemia (MLL-ALL) xenografts, demonstrating that p53 and reduction of asparagine cooperate in tumor inhibition [314].

PHGDH, the enzyme that catalyzes the first reaction in the *de novo* serine synthesis pathway from 3PG, is overexpressed in breast carcinomas and melanomas [25, 26]. Although the mechanisms whereby PHGDH exerts oncogenic effects remain unclear, PHGDH is a potential target for the development of novel anticancer drugs. RNAi based strategies against this enzyme have been tested, however, its inhibition fails to affect serine availability (Figure 5) [25, 26].

### Krebs cycle and oxidative phosphorylation

PDK1 is a gate-keeping mitochondrial enzyme that regulates the flux of pyruvate into the mitochondria. PDK isoforms are remarkably overexpressed in multiple human tumor samples and they show low expression in normal tissues, which may minimize side effects of this enzyme inhibition [315]. Dichloroacetate (DCA) is a structural analog of pyruvate and inhibits PDK1, changing the cancer cells metabolism of cancer cells from cytoplasm-based glycolysis to mitochondria-based glucose oxidation (Figure 5) [316]. DCA indirectly stimulates the activity of pyruvate dehydrogenase (PDH), which is hyperactivated by Myc, RTK or HIF1 signaling [317]. DCA has been used in combination with other drugs in breast, ovarian, pancreatic and colorectal cancer [318]. DCA can alter the mitochondrial membrane potential in many cancer models, showing increased production of ROS and decreased efflux of proapoptotic mediators from the mitochondria [319]. DCA is able to increase extracellular pH by reducing lactate secretion [320], thus limiting local invasion [9]. Due to its low price and toxicity and long history of clinical application, this drug serves as a potential metabolic-targeting molecule for sensitizing cancer cells, especially in glioblastoma [321]. A study has shown that DCA is able to increase the transcriptional activity of p53 in cancer stem cells (CSCs), inducing Bax-depended apoptosis [322].

Isocitrate dehydrogenase (IDH) of the TCA cycle is an essential enzyme for mitochondria respiration. Different IDH inhibitors have been developed with encouraging *in vitro* efficacy. An IDH1 inhibitor, called HMS-101, was able to block colony formation of leukemia cells [323], while others like AG-120, AG-881 and IDH305 went to phase I of clinical development (Figure 5) [324]. A cancer clinical trial involving patients with cancer presented promising preliminary results [325].

Cytosolic malic enzyme (ME1) catalyzes the reversible oxidative decarboxylation of malate to pyruvate, generating carbon dioxide and NADPH and contributing for macromolecules biogenesis [326]. Pre-clinical studies targeting ME1 have shown positive effects on cancer cells, but as far as we know, no clinical therapy targeting inhibit ME1 have yet been approved [327, 328].

Metformin (1,1-dimethylbiguanide) was discovered in 1920's and is derived from the alkaloid galegine or isoamylene guanidine, an active substance of *Galega officinalis* [329]. Belonging to the biguanide class of antidiabetic drugs, metformin is the most commonly prescribed therapy for type 2 diabetes patients [330]. Indeed, it has a broad use, including polycystic ovarian syndrome, metabolic syndrome and diabetes prevention. Increased glucose consumption is a hallmark of most cancer cells, and increased blood glucose and insulin levels observed in type 2 diabetes are associated with poor cancer prognosis [331]. Studies have shown that cancer mortality was substantially reduced in diabetic patients treated with metformin compared to other treatments [332, 333], bringing metformin to the spotlights of cancer therapy research.

Metformin acts directly in the reduction of insulin resistance and blood glucose concentration without causing hypoglycemia. Its mechanism of action is only now becoming clear, although it was first introduced in 1957 in Europe and in 1995 in USA [334]. This natural compound may suppress tumor progression by modulating whole body metabolic physiology or by acting directly in cancer cells, inducing a condition similar to caloric restriction [335]. Metformin impairs malignant growth indirectly by reduction of systemic glucose and insulin levels and directly by suppressing mTOR signaling, mitochondrial glucose oxidation and/or reducing stability of HIF under hypoxic conditions [336].

The first identified metformin cellular target in cancer came from the discovery of LKB1, a major upstream activator of AMPK [337]. The study has shown that metformin acts as a growth inhibitor in a dose-dependent manner by suppressing the mTOR/S6K pathway via LKB1–AMPK interaction in breast cancer cells, being the first to demonstrate its anticancer activity. LKB1-dependent and AMPK-dependent suppression of the mTOR pathway are possibly the most potent antineoplastic effects of metformin. Some types of cancers may have inactive LKB1 and defects in the LKB1–AMPK pathway, which may potentiate the risk of metabolic transformation of pre-neoplastic cells [338].

Another described mechanism of action of metformin besides LKB1 is through inhibition of the mitochondrial electron transport chain complex I [339]. This inhibition interrupts mitochondrial respiration and decreases proton-driven synthesis of ATP, causing a cellular energetic stress state and elevation of the AMP:ATP ratio. These changes result in allosteric activation of AMPK, a major sensor of cellular energy status. Therefore, metformin decreases growth signaling due to increased AMP:ATP ratio [340]. Metformin also diminishes glucose transport by inhibiting HK2 [339].

Through inhibition of mitochondrial complex I, metformin also reduces production of reactive oxygen species, oxidative stress and DNA damage. A recent study suggested that inhibition of mitochondrial glycerophosphate dehydrogenase (mGPD) could be the primary mechanism of metformin-mediated inhibition of gluconeogenesis [341]. Furthermore, metformin is able to inhibit both EMT and OxPhos markers, inducing a striking inhibition of proliferation and colony formation of acidic melanoma cells [342] and decreasing glucose oxidation through glutamine metabolism in prostate cancer cells [343].

Besides its action in mitochondrial complex I and LKB1, metformin has presented other possible targets. Many investigations suggest direct targets like ATM and Ragulator and several indirect targets like PKA, c-Myc, DICER, p53/REDD1 and NF-κB, revealing different mechanisms of action in cancer prevention and therapy [344]. Metformin is also able to decrease IGF1–insulin receptors signaling by lowering insulin levels and AMPK-dependent phosphorylation of IRS1 and to decrease mTORC1 signaling by inactivating Ragulator, mimicking the effects of amino acid starvation [344, 345]. A recent study described how p53/REDD1 axis causes mTORC1 downregulation through AMPK-independent mechanisms. In prostate cancer cell lines with wild-type p53, metformin may upregulate REDD1-mediated mTORC1 inhibition and cell-cycle arrest [346].

Several studies point the important relationship between metformin and p53 and highlight how p53 participates in metformin mechanisms of action, especially for cancer treatment. One study pointed a negative relation between metformin and p53, showing that AMPK activation by metformin diminishes p53 protein levels and oxidative stress [347]. The same study also has shown that metformin increases the deacetylation of p53 at a SIRT1-target site, which may turn it more susceptible to MDM2-mediated degradation. However, other studies have shown a positive correlation. In breast cancer, p53 is required for metformin-induced cell growth inhibition, further consolidating metformin use as an antitumor strategy [348]. Another study has shown that exposure of HepG2, a hepatoma cell line, to low doses of metformin results in induction of cell senescence, through AMPK pathway, in a p53-dependent manner [349]. As already described, the combination of metformin and 2DG selectively enhanced cytotoxicity of doxorubicin against MCF-7 cells through p53 [249]. The knowledge coming from research regarding metformin mechanism of action pathways currently allows the development of multiple therapeutic strategies for cancer treatment.

### Cell growth signaling

Epidermal Growth Factor (EGF) is a well-known growth factor that is overexpressed in many types of cancer. Thus, EGF receptor (EGFR) family has been effectively targeted using drugs such as lapatinib (tyrosine kinase inhibitor active against EGFR and HER2) and trastuzumab (a humanized antibody targeting the HER2 receptor). Evidences suggest that these agents may be useful for clinical purposes, although studies suggest that they may increase resistance to other chemotherapeutics [350]. A study has shown that lapatinib downregulates and destabilizes mutant p53 via modulation of HSF1 activity in HER2-positive breast cancer cells, suggesting therapeutic benefits of the inhibitor [351]. Another one has shown that p53 expression could predict the complete response to chemotherapy in patients with HER2-positive breast cancer treated with sequential cycles of doxorubicin and cyclophosphamide followed by trastuzumab and paclitaxel [352].

mTOR is another well-known protein that is overexpressed in several types of tumors. It is regulated by nutrient availability and its activation stimulates a metabolic program to promote cell growth [201]. Rapamycin is a drug that inhibits the mTOR complex 1 (mTORC1) and is able to enhance the antitumor effects of cisplatin in gastric cancer (Figure 5) [353]. Data also suggest that mTOR inhibition with a dual PI3K/mTOR inhibitor, NVP-BEZ235, may revert chemoresistance in other types of cancer [354]. Furthermore, rapamycin increases PUMA expression and PARP cleavage, suggesting that MDM2 suppression by rapamycin stimulates p53-mediated apoptosis [355]. Rapalogues are any chemical agents that resemble rapamycin in its capacity to inhibit mTOR enzymatic activity. The therapeutic benefit provided by mTOR inhibitors may be limited by the intrinsic ability of these compounds to stimulate autophagy and hence render established tumors resistance to chemotherapeutics [356]. Inhibitors of mTOR downstream targets, like S6K, also presented positive effects against cancer. PF-4708671, a specific inhibitor of S6K1, is able to reduce proliferation of prostate, breast and lung cancer cells [199, 357, 358].

Hypoxia-inducible factor 1 (HIF1) is a transcription factor that controls the synthesis of several glycolytic enzymes and angiogenic factors in response to hypoxia and other stress conditions, increasing glucose uptake and its oxidation to lactate in order to generate ATP through an oxygen-independent mechanism [265]. T-lymphoblastic leukemia cell lines treatment with a HIF1 inhibitor called echinomycin inhibits cancer cells growth [359]. HIF1 inhibitors, such as acriflavine (ACF) and PX-478, have generated promising results in pre-clinical studies, yet have not entered clinical development [360, 361]. ACF is able to increase the expression of p21, and reverse the phosphorylation of CDK2 in a p53 dependent mechanism [362]. Inhibition of HIF-1α by PX-478 occurs in both normoxia and hypoxia and does not require p53 [363].

The phosphatidylinositol 3-kinase (PI3K) is a lipid kinase and an upstream activator of AKT and mTOR, as previously mentioned (Figure 5). This axis is one of the most commonly deregulated signaling networks in human cancers [364], since it promotes survival, glycolytic metabolism, fatty acid synthesis and cell growth mechanisms [4]. The therapeutic perspectives related to the PI3K pathway has been recently reviewed [365]. p53 induction is blocked by the PI3K inhibitor LY294002, implicating that the PI3K pathway is a critical mediator of p53 activation. Besides, LY294002 is able to inhibit p53 stabilization and functional activation in a variety of cell types and in response to several different DNA-damaging agents [366]. PI3K pathway inhibition can increase the sensitivity to adriamycin in HER-2/neu expressing breast tumor cells in a p53 dependent way [367]. GDC-0941 and PX866 are two drugs that inhibit PI3K currently in clinical trials (ClinicalTrials.gov identifiers: NCT00876109, NCT00726583). Data suggest that advanced solid tumors, like metastatic breast and non-Hodgkin's lymphoma, are the most promising targets of those compounds [270].

## THERAPY TARGETING P53

The ability of p53 to induce cell death has been thoroughly reviewed [368, 369]; however, its impact in metabolism is less discussed, even though it has an impact in cancer therapy. Here, we make a comprehensive review of how different therapies that induce p53 expression impact tumor cell metabolism. More specifically, how these therapies modulate key metabolic genes and how metabolism may dictate whether the cell undergo senescence, autophagy or apoptosis and more importantly resistance to chemotherapy.

### Inducers of p53

As a general stress sensor protein, p53 is induced by DNA damage and several conventional anticancer therapies cause DNA damage, the most relevant in cancer therapy are ionizing radiation (IR) and genotoxic drugs.

### Ionizing radiation

More than 50% of the cancer patients are treated with radiotherapy [370]. Ionizing radiation generates ROS causing DNA damage, cell death and recruitment of immune cells to the tumor site [371]. Ionizing radiation induces apoptosis and senescence of fibroblasts, which show phosphorylation of p53 and increased p21 expression, DNA damage was evident and senescence markers are evident on the first day of IR and progressively increases [372]. However, senescent tumor associated fibroblasts induced by low doses of IR express high levels of metalloproteinases (MMPs), which can favor malignant lung cancer cells growth *in vitro* and *in vivo*, indicating that senescent cells may enhance the growth of adjacent malignant cells [373].

**Figure 6:**
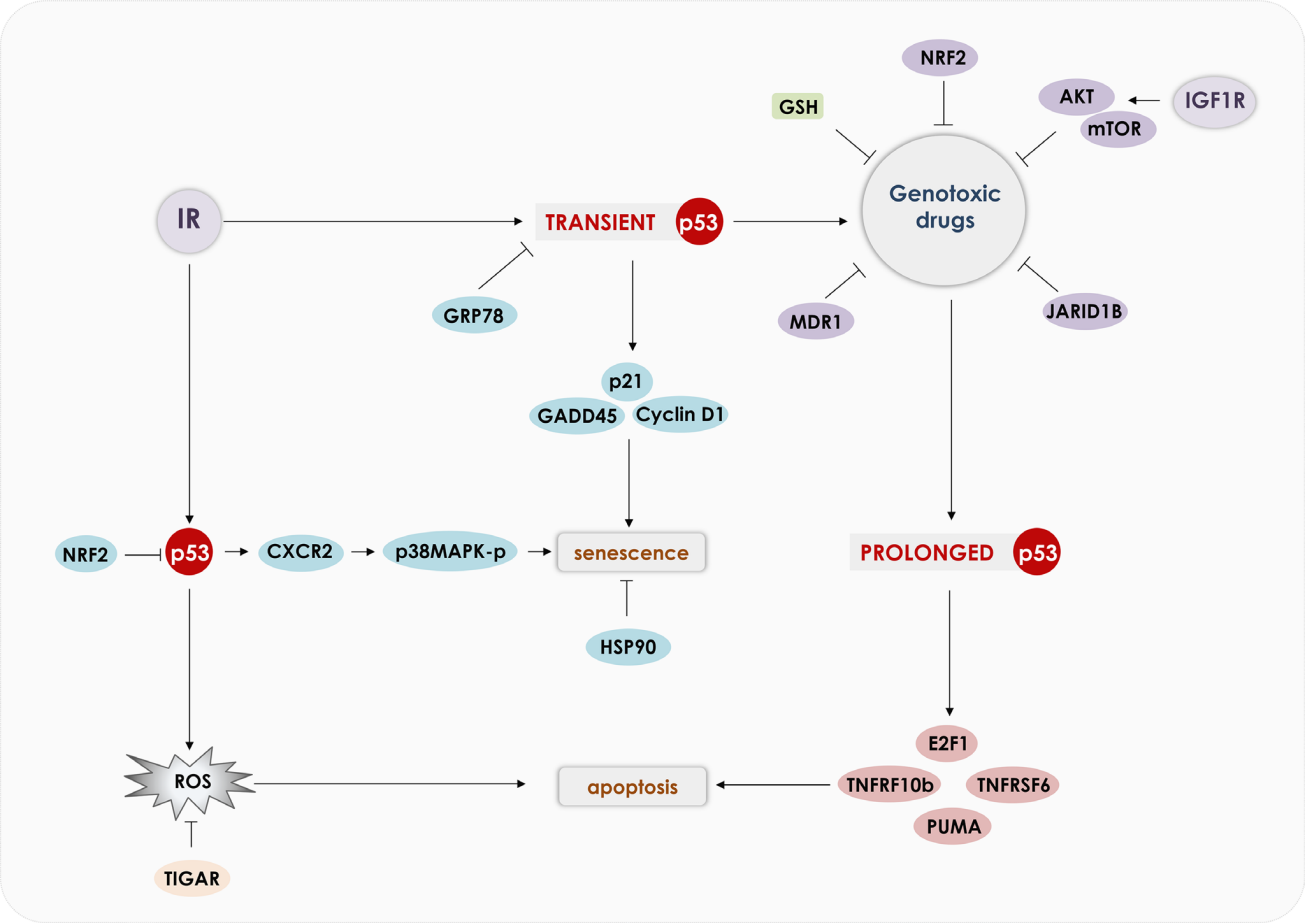
Mechanism of action of p53 inducers Ionizing radiation (IR) induces p53, which increases ROS levels and induces apoptosis. TIGAR and NRF2 may prevent this activity. p53 induction by IR may also induce CXCR2, which induces phosphorylation of p38MAPK and senescence. Genotoxic drugs may have their activities modulated by NRF2, GSH, MDR1, JARID1B and the AKT/mTOR pathway, which is induced by IGF1R. Low drug concentration induces transient p53 expression and the target genes: p21, cyclin D1 and GADD45 that directs the cell to senescence, which can be inhibited by HSP90. Prolonged p53 expression induced by genotoxic drugs is involved in upregulation of E2F1, and the pro-apoptotic genes: TNFRSF10b (Killer-Dr5), TNFRSF6 (Fas-Apo) and PUMA.

Tumors derived from cells expressing wild-type p53 are more sensitive to gamma radiation, compared to cells with mutations in the p53 gene [374, 375]. Treatment with ionizing radiation induced senescence of Glioblastoma (GBM) cells expressing wild-type p53, but not cells with mutant p53 (T98) or cells expressing HPV E6 protein [376]. PTEN transcription is regulated by p53 as mentioned before [122, 377], and its status is important to determine IR effect. IR triggered apoptosis in PTEN positive cells, while in PTEN negative cells treatment led to AKT activation and high levels of ROS, which were essential to induce senescence. Interestingly, depletion of p53 prevented IR induced senescence in these cells [378]. p53 status is important to determine induction of senescence or cell death mediated by IR, as cells expressing mutant p53 had delayed and persistent induction of p21 after IR [379]. Senescence mediated by IR depends on p53 induction of CXCR2, which is important to induce phosphorylation of P38MAPK and senescence [380]. Apoptosis induced by IR is increased by knockdown of NRF2, showing the importance of ROS induction also in the apoptotic process. Autophagy mediated by IR showed an anti-apoptotic activity through induction of MAPK 1/2 phosphorylation and NRF2 upregulation. NRF2 mediated p53 inhibition and induced p65 and Bcl2 that exerted the anti-apoptotic activity (Figure 6) [381].

### Genotoxic drugs

p53 rapidly responds to DNA damage, promoting activation of cell cycle mediators, regulating metabolism and giving time to the DNA repair machinery to fix the damage, however, if the DNA damage is too extensive it will promote cell death. Several drugs cause DNA damage and p53 activation is one of the key events to trigger their actions.

Anthracyclines intercalate with DNA, inhibiting replication and transcription, they also interact with topoisomerase II leading to DNA breakage [382]. Epirubicin is an anthracycline, which induces TIGAR in a p53 dependent manner. Even though TIGAR is important to inhibit glycolysis, it also can reduce epirubicin toxicity, by reducing ROS levels, autophagy and apoptosis [383],showing that TIGAR induction by p53 can have pro-survival activity [384, 385]. The DNA damaging agents: etoposide and the anthracycline doxorubicin target OxPhos, promoting mitochondrial biogenesis, increasing ROS and inducing apoptosis, which are reduced by p53 deficiency [386]. Drug concentration is important to determine senescence or apoptosis. Low concentrations of doxorubicin induced senescence with increased genomic instability and increased expression of p53, p21 and Cyclin D1 [387]. Also, p21 was shown to be important to induce senescence and at the same time to reduce apoptosis mediaed by IR and doxorubicin [388]. Events underlying senescence mediated by doxorubicin are increased ROS levels, transient p53 activation, sustained p21 and ATM/ATR activation; while low and prolonged p53 and E2F1 upregulation with absence of p21 occurred in apoptotic cells [389]. Therefore p53 and p21 act as positive regulators of senescence, but are also not sufficient or absolutely required for doxorubicin mediated senescence [390]. Doxorubicin induced senescence dependent on p53 and p21 activation can be accelerated by inhibition of Heat shock protein 90 (HSP90), a chaperone that participates in DNA damage response [391]. Therapies that inhibit HSP90 may increase doxorubicin activity [391]. During senescence or apoptosis there is a different expression pattern mediated by p53. In normal senescent fibroblasts p53 can be found in the promoter of p21 and GADD45; after DNA damage mediated by doxorubicin, p53 binds to promoters of apoptotic genes like TNFRSF10b (DR5), TNFRSF6 (Fas-Apo) and PUMA [392].

Cisplatin (CPPD) is used in cancer therapy since 1971, it forms adducts with DNA and inhibits replication, transcription and cause DNA damage. It is used for treatment of several types of cancer; however, development of drug resistance poses as a major limitation for its wider use [393]. Cisplatin induced senescence in hepatoma cells is dependent on p53 and p21 activation and ROS induction [394, 395]. Different players modulate CPPD-induced senescence.IGF1R can promote proliferation and chemotherapy resistance and its inhibition increases p53 mediated apoptosis induced by CPPD and reduces senescence [396]. Glucose-regulated protein 78kda (GRP78), a chaperone localized in the ER, is an important inhibitor of senescence mediated by CPPD, responsible for reduced p53 expression and Ca2+ release inhibition from endoplasmic reticulum [397].

Drug resistance to CPPD is an important issue in the clinic, and is correlated to absence of apoptosis and is not due to autophagy, in fact pro-autophagic stimuli, such as inhibition of mTOR or AKT, induce cell death [398]. Rapamycin-insensitive companion of mTOR (Rictor) is a component of mTORC2, it is downregulated by CPPD in ovarian cancer cells and its knockdown in chemo-resistant cell lines sensitizes the cells to apoptosis dependent on p53 status, showing that mTOR pathway may be important in resistance to cisplatin [399]. Cells resistant to cisplatin were shown to be more sensitive to Nutlin, a drug responsible for disruption of MDM2 and p53 interaction, leading to p53 stabilization [158]. CPPD resistant cell lines also show increased activity of IGF1R/AKT signaling. Inhibitors of IGF1R or AKT induced apoptosis in CPPD-resistant cell lines in combination with Nutlin-3 by increasing p53 levels and inhibiting pro-survival autophagy [400]. UT-SSC26A is a cell line with enhanced chemoresistance to CPPD, it expresses a truncated p53 [401]. These cell lines have an upregulated expression of ABC transporters (ABCC2 and ABCG2), increased metabolic activity and GSH levels. Several chemotherapeutic agents, including CDDP, doxorubicin, methotrexate and vincristine can form conjugates with glutathione, which can be exported out of the cell by ABCC2, therefore inhibition of the ABC transporters recovers sensitivity to CPPD [402]. GSH may act not only as a cofactor in MRP2-mediated CPPD efflux, it may as well have a redox-regulating cytoprotective activity and function as a copper chelator [403].

JARID1B is a histone H3K4 demethylase [404]. It is interesting that multi-resistant cells show slow cycling properties, increased OxPhos and increased expression of JARID1B; direct knockdown of JARID1B or inhibition of mitochondrial respiration blocked the JARID1B subpopulation and sensitized the cells to chemotherapeutic drugs [405]. JARID1B has also been shown to inhibit p53 expression and JARID1B inhibition suppressed proliferation and invasion [406]. Expression levels of JARID1B were positively correlated with chemotherapy resistance in ovarian cancer patients [407].

As mentioned before mutant p53 has been shown to induce MDR1. Mutant p53 induces expression of MDR1 also through NF-κB upregulation, increased P-gp activity and showed increased resistance to doxorubicin [408]. Another gene involved in chemoresistance to cisplatin is NRF2. It was shown that mutant p53 increases NRF2 levels, which increases Bcl2 and Bcl-x, showing an unfavorable outcome after treatment with CPPD [105]. It is interesting that it had been observed that overexpression of either wild-type p53 or mutant p53 was shown to increase resistance to cisplatin, in this case cytoplasmic p53 inhibited caspase 9 activity and reduced chemo sensitivity [409]. Figure 6 shows how different agents may inhibit IR and genotoxic drugs activity and how different levels of p53 induces senescence or apoptosis.

### Inhibitors of MDM2

MDM2 was firstly shown to interact with p53 and inhibit its transcriptional activity [410]. Their interaction happens through their N-terminal regions [411], on p53 at the residues F19, W23 and L26 [412]. The protein levels of MDM2 and p53 are tightly linked, as p53 activates the expression of MDM2 [413], which in turn acts as an E3 ubiquitin ligase, responsible to drive p53 degradation through proteasome, creating an auto regulated feedback loop [414, 415]. MDM2 induces ubiquitination of lysine residues in the p53 C-terminal region and alteration of these lysine residues to arginine generated a protein with high transcriptional activity and resistant to MDM2-mediated degradation [416]. Nutlins are cis-Imidazole molecules that mimic the p53 residues F19, W23 and L26 and have been shown to interact and inhibit MDM2 [417]. Some Nutlin derived compounds have reached clinical trials, RG7112 was developed by Roche and was tested in phase I clinical trials for different types of cancers, including, liposarcoma, soft tissue sarcoma, myelogenous leukemia and hematologic neoplasms, resulting in activation of p53, p21 and apoptosis induction [418, 419]. As mentioned before, its combination with asparaginase resulted in stronger antitumor activity [314].

Combination of Nutlin-3 and PI-103 (inhibitor of the PI3K/AKT/mTOR pathway) increased Bax activation, caspase-3 cleavage and apoptosis of AML cells, even though induction of several p53 target genes, like p21, NOXA, Bcl2, MDM2 and Survivin were reduced due to mTOR inactivation and attenuation of p53 protein synthesis. Nutlin-3 also cooperates with PI-103 in the blockage of p70S6K and 4E-BP1 phosphorylation, which also contributed to the reduction in protein synthesis [420]. Mantle cell lymphoma (MCL) cells with wild-type p53 status treated with only Nutlin-3 showed increased levels of p53 and activated p53 (p-ser15-p53), which induced phosphorylation of AMPK and decreased phosphorylation of rpS6, 4E-BP1, p70S6K and AKT. Cells with p53 mutated status and treated with Nutlin-3 did not show the same trend [421]. The intensity of p53 induction had different impacts in the modulation of the mTOR pathway [422]. This differential activity may modulate senescence dependent and independent of mTOR.

Treatment with Nutlin-3 induced cellular quiescence of human fibrosarcoma cells and non-tumorigenic human fibroblasts [423], inhibiting the phosphorylation of rpS6 and 4E-BP1, and combination of rapamycin and Nutlin-3a also suppressed senescence [424]. This suppression is mediated by TSC2, a p53 target gene that negatively regulates mTOR, indicating how p53 can inhibit mTOR and prevent mTOR mediated senescence [425]. In melanoma cell lines Nutlin-3a failed to inhibit mTOR and the cells underwent senescence, which could be converted to quiescence by combining Nutlin-3a and Rapamycin, which inhibited mTOR [425].

Glioblastoma multiforme (GBM), renal carcinoma and breast cancer cells treated with Nutlin-3 entered senescence in a mechanism that may be dependent on active mTOR [426–428]. Adult T-cell leukemia (ATL) cells treated with Nutlin-3 also undergo senescence, even in the absence of p16INK4a and p14ARF, important regulators of senescence. The p53 target genes, p21, PIG3 and TIGAR were induced by Nutlin-3, knockdown of TIGAR reduced apoptosis and senescence mediated by Nutlin-3, indicating that TIGAR may also be a mediator of senescence [429]. In leukemic cells Nutlin-3 was shown to down-regulate E2F-1 [430], which was shown to inhibit cellular senescence [431], indicating another pathway for senescence induction mediated by Nutlin-3. Nutlin-3 combined with metformin showed increased induction of senescence. Metformin up-regulated p53 and increased phosphorylation of AMPK and decreased in phosphorylation of mTOR [348]. Combination of Nutlin-3 and IR induced senescence of lung cancer cells [432].

Overall, the data from the literature indicate that mTOR plays an important role in senescence induction, and p53 modulation is an essential component in different therapies for the outcome of cell quiescence or senescence. High levels of p53 inhibit mTOR and prevent senescence, while medium levels of p53 do not inhibit mTOR and induces senescence in some cell lines [140, 422], while in other cell lines p53 activation induced senescence through different mechanisms [429, 431].

Nutlins can also induce autophagy. It has been suggested that autophagy inhibits apoptosis and promotes survival in cells treated with Nutlin [158]. However, other reports indicate that autophagy induced by Nutlin-3a can be pro-apoptotic and dependent of AMPK activation, which promoted autophagy through phosphorylation of ULK1 [433]. The pro-apoptotic autophagy was shown to be dependent on p53 positive status [434]. Metabolism plays an essential role in protective autophagy. Nutlin sensitive cells show increased activation of AMPK, induction of ROS, inhibition of glycolysis, mTOR and autophagy. Glycolysis plays a role to limit ROS induction, regulating expression of ATG and favoring autophagic flux. Therefore in cell lines that p53 do not limit glycolysis there is an increased autophagic flux and resistance to apoptosis [158]. ROS has also been shown to inhibit autophagy by decreasing ULK1 expression, which is induced by p53 after phosphorylation (S392) mediated by p70S6K [161]. Oxidants seem to play an essential role in the inhibition of autophagy and induction of senescence and apoptosis.

### Modulators of mutant p53

Prima1 and Prima1-met (APR-246) are small molecules that rescue mutant p53 activity [435, 436], there are already early phase 1 clinical trials using Prima1-met for Esophageal carcinoma, acute myeloid leukemia, myelodysplastic syndrome and myeloproliferative neoplasm (ClinicalTrials.gov identifiers: NCT02999893, NCT03072043).

Prima1 and CP-31398 have shown chemopreventive activity by modulating p53 in tobacco carcinogen-induced lung adenocarcinoma model [437]. Proteomic analysis of breast carcinoma cell lines expressing mutants p53A278P, p53R280K or p53M385T treated with Prima1-met, showed differential expression of Annexin A1, Annexin A2, bifunctional methylenetetrahydrofolate dehydrogenase (MTHFD2), L-lactate dehydrogenase (LDH), GAPDH, malate dehydrogenase (MDH2) and voltage-dependent anion-selective channel protein 2 (VDAC2). GAPDH, LDH, MTHFD2, and MFH2 are responsible for regulating glycolysis progression, but are also linked to apoptosis, as apoptosis is ATP dependent, demanding high energy levels. Annexins were associated with activation of caspases, p38, and JNK signaling, while VDAC2 is associated with mitochondrial permeabilization, indicating that Prima1-met is able to activate genes involved in cell death and metabolism [438]. Prima1-met was also shown to be associated with down-regulation of O6-methylguanine-DNA methyltransferase (MGMT), which removes methyl adducts from guanine and is a major determinant of resistance to alkylating agents. In a glioblastoma cell line, low levels of Prima1-met induced senescence, and high concentrations induced massive cell death [439]. Prima 1 induced degradation of mutant p53 and autophagy in cell lines expressing wild type or mutant p53 [440, 441].

Hypoxia increases Prima-1 activity in breast cancer cells expressing mutant p53, possibly through oxidative stress, indicating that Prima-1 may prevent hypoxia-mediated chemoresistance [442]. The acute myeloid cell line KBM3 with mutant p53 treated with Prima1-met presented increased ROS levels, causing activation of heme oxygenase-1 (HMOX1) mediated by NRF2. Inhibitors of PI3K (wortmannin) and mTOR (rapamycin) prevented upregulation of HMOX1, increasing antitumor effects mediated by Prima1-met [443]. Prima1-met depleted GSH and induced ROS, independently of p53 [444]. ROS induction can also be achieved by modulation of TrxR1, an important enzyme that catalyzes thioredoxin that can be modulated by Prima1-met to exert a NADPH oxidase activity, an inductor of ROS, indicating one possible mechanism of how Prima1-met can induce ROS in a p53 independent manner [445]. Besides TrxR1 inhibition, Prima1-met may also inhibit other antioxidant enzymes, like Prx3 and GPx-1 [446]. Prima1 can have antitumor effects in cells with different p53 status, indicating that it has other mechanisms of action independent of mutant p53, but p53 status may modulate different outcomes, inducing apoptosis or necrosis [447, 448].

Mutant p53 have been shown to induce chemoresistance as discussed before. Prima1-met is also able to restore sensitivity to cisplatin and doxorubicin and 5-FU resistant cell lines expressing mutant p53. Its active form methylene quinuclidinone is able to directly interact and inhibit glutathione [449, 450] and prevents GSH mediated drug inhibition [402, 403].

### p53 gene therapy and metabolism

In the early 1990s direct transfer of the p53 gene showed strong anti-tumor effects [451–456]. Soon viral vectors were employed to transfer p53 gene in clinical trials for patients with different types of cancer, using retroviral [457] or adenoviral vectors expressing p53 [458, 459]. Different adenoviral vectors expressing p53 (Advexin, Gendicine and SCH58500) have been tested in clinical trials, but only Gendicine is regularly used in the clinics [458, 460–461]. Differently from the approaches described before, this technique allows direct expression of the wild-type p53, without off target effects. At the same time, it can have strong antitumor effects even in cells with mutant or negative p53 status.

Transfer of the p53 gene conferred strong anti-tumor activity to a conditionally replicating adenovirus, this oncolytic adenovirus suppressed expression of p21 and showed strong induction of autophagy through DRAM activation [462]. ROS plays an essential role in p53 mediated cell death. First generation Adenovirus-p53 (Ad-p53) activates cell death through ROS induction and GSH depletion, which could be enhanced by combination with 2-deoxyglucose. This apoptotic activity can be partially inhibited by adenoviral vectors expressing catalase or glutathione peroxidase [463, 464]. Bcl-XL expression also prevented generation of ROS and inhibited induction of senescence mediated by an Ad-p53 [465]. Ad-catalase was shown to inhibit apoptosis mediated by DNA damaging agents; catalase increased p53 degradation and decreased its phosphorylation at serine 20 [466]. Higher levels of p53 mediated by an auto-regulated promoter induced ROS, that participated in prostate cancer cell lines apoptosis, even in p53 negative cell lines [94].

Ad-p53 has also been shown to increase drug sensitivity to 5-FU, doxorubicin, cisplatin, methotrexate, paclitaxel, carboplatin and induce cell death in chemoresistant cell lines. This effect is the result of the reduced expression of MDR1 [467–470]. It´s interesting to highlight that an Ad-p53 had a stronger effect in cisplatin and doxorubicin drugs resistant cell lines compared to sensitive cell lines. Drug resistant cell lines displayed higher transduction efficiency though [471].

Other p53 target genes have also been employed, like Ad-Puma, which induced ovarian cancer cells death, generating ROS, but as a protective mechanism also activated the NRF2/HMOX1 pathway [472]. Ad-p21 is capable to induce senescence, nevertheless, cells expressing p53 show increased levels of ROS and apoptosis, and the magnitude of ROS is important to determine cell fate between senescence and apoptosis [473].

## CONCLUDING REMARKS

Like Janus, p53 has two faces, may be an oncogene or a tumor suppressor. In the beginning its mutant form rendered it an oncogenic function. However, wild-type p53 was revealed and aroused the notion of the tumor suppressors, turning p53 into the guardian of the genome. This transcription factor regulates genes involved in almost all hallmarks of cancer. Despite the fact that modulation of metabolism is one of the most underestimated activities of p53, it is a key factor to promote tumor survival, playing an important role in senescence, autophagy, apoptosis and resistance to chemotherapy.

## SUPPLEMENTARY MATERIALS TABLE





## Abbreviations

2PG: 2-phosphoglycerate
3PG: 3-phosphoglycerate
3PO: 3-(3-pyridinyl)-1-(4-pyridinyl)-2-propen-1-one
3PHP: 3-phosphohydroxypyruvate
ALDH4: aldehyde dehydrogenase 4
AKT: AKT serine/threonine kinase
ASS1: argininosuccinate synthetase 1
ASNS: asparagine synthetase
ATG12: Autophagy Related 12
ACLY: ATP citrate lyase
BPTES: Bis-2-(5-phenylacetamido-1,2,4-thiadiazol-2-yl) ethyl sulfide
BCL2: BCL2, apoptosis regulator
PUMA: BCL2 binding component 3
BAX: BCL2 associated X, apoptosis regulator
BECN1: beclin 1
MTHFD2: bifunctional methylene tetrahydrofolate dehydrogenase
BRCA1: BRCA1, DNA repair associated
CPT1C: carnitine palmitoyltransferase 1C
CPPD: Cisplatin
p21: cyclin dependent kinase inhibitor 1A
p16INK4a/p14ARF: Cyclin Dependent Kinase Inhibitor 2A
COX: cytochrome C oxidase complex
SCO2: cytochrome C oxidase 2
CXCR2: C-X-C Motif Chemokine Receptor 2
P5C: Delta-1-pyrroline-5-carboxylate
DRAM1: DNA Damage Regulated Autophagy Modulator 1
E2F-1: E2F Transcription Factor 1
EGF: Epidermal Growth Factor
TNFRSF6: Fas cell surface death receptor
EGCG: epigallocatechin gallate
FASN: Fatty acid synthase
FDG: fluoro-2-deoxy-glucose
F16BP: fructose-1,6-bisphosphate
F26BP: fructose-2,6-bisphosphate
GOF: gain of function
G6PDH: glucose 6-phosphate dehydrogenase
GDH: glutamate dehydrogenase
GLS: glutaminase
GSH: glutathione
GPX1: glutathione peroxidase
HSP90: Heat shock protein 90
HMOX1: Heme Oxygenase 1
HIF-1: hypoxia-induced factor 1
IGFs: insulin-like growth factors
IDH1: isocitrate dehydrogenase
LPIN1: lipin 1
LDH: L-lactate dehydrogenase
JARID1B: Lysine Demethylase 5B
MLL1: Lysine Methyltransferase 2A
MLL2: Lysine Methyltransferase 2D
MOZ: Lysine Acetyltransferase 6A
ME1: malic enzyme 1
MCD: malonyl CoA decarboxylase
MDH2: malate dehydrogenase
mTOR: mechanistic Target Of Rapamycin kinase
MDM2: MDM2 proto-oncogene
MAPK: mitogen-activated protein kinase 1
Mn-SOD: Mn superoxide dismutase
MCT4: Monocarboxylate transporter 4
NOX1: NADPH oxidase
MDR1: Multi-drug resistance
NRF2: NF-E2 related factor 2
OxPhos: oxidative phosphorylation
PANK1: panthothene kinase 1
PGC-1α: Peroxisome proliferator-activated receptor γ Coactivator 1-α
PET: Positron Emission Tomography
NOXA: phorbol-12-myristate-13-acetate-induced protein 1
PTEN: phosphatase and tensin homolog
PI3K: phosphatidylinositol-4,5-bisphosphate 3-kinase catalytic subunit beta
PEP: phosphoenolpyruvate
PHGDH: phosphoglycerate dehydrogenase
PGAM1 or PGM1: phosphoglycerate mutase 1
POX: proline oxidase
PFK-1: proline dehydrogenase Phosphofructokinase
PRODH: proline dehydrogenase
PIG6: p53-induced gene 6
PIG3: p53-induced gene 3
PYCR1: pyrroline-5-carboxylate reductase
PDK2: pyruvate dehydrogenase kinase 2
PK: pyruvate kinase
PKM2: pyruvate kinase M2
Rb: Retinoblastoma transcriptional corepressor 1
ROS: reactive oxygen species
S6K1: ribosomal protein S6 kinase B1
p65: relA Proto-Oncogene, NF-KB Subunit
SESN: Sestrin
GLUT1: Solute Carrier Family 2 Member 1
Glut 4: Solute Carrier Family 2 Member 4
TIGAR: TP53-induced glycolysis and apoptosis regulator
TRAIL: TNF Superfamily Member 10
DR5: TNF receptor superfamily member 10b
TrxR1: Thioredoxin Reductase 1
TKTL1: Transketolase-like protein 1
TCA: tricarboxylic acid
TSC2: Tuberous Sclerosis 2
ULK1: unc-51 like autophagy activating kinase 1
VDAC2: voltage-dependent anion-selective channel protein 2
