# Correction: p53 and metabolism: from mechanism to therapeutics

**DOI:** 10.18632/oncotarget.26181

**Published:** 2018-09-21

**Authors:** Fernando M. Simabuco, Mirian G. Morale, Isadora C.B. Pavan, Ana P. Morelli, Fernando R. Silva, Rodrigo E. Tamura

**Affiliations:** ^1^ Laboratory of Functional Properties in Foods, School of Applied Sciences (FCA), Universidade de Campinas (UNICAMP), Limeira, São Paulo, Brazil; ^2^ Center for Translational Investigation in Oncology/LIM24, Instituto do Câncer do Estado de São Paulo (ICESP), São Paulo, Brazil; ^3^ Department of Radiology and Oncology, Faculdade de Medicina, Universidade de São Paulo, São Paulo, Brazil; ^4^ Universidade Federal de São Paulo, Departamento de Ciências Biólogicas, Diadema, SP, Brazil

**This article has been corrected:** The correct author affiliation is given below:

**Rodrigo E. Tamura**^4^

^4^ Universidade Federal de São Paulo, Departamento de Ciências Biólogicas, Diadema, SP, Brazil

Original article: Oncotarget. 2018; 9:23780-23823. https://doi.org/10.18632/oncotarget.25267

