# Supplementary material for: p53 and metabolism: from mechanism to therapeutics
Source: Oncotarget. 2018 May 4;9(34):23780–823. doi: 10.18632/oncotarget.25267 (PMC5955117; doi:10.18632/oncotarget.25267)
Supplement: Supplementary file 1 [file oncotarget-09-23780-s001.pdf]

p53 and metabolism: from mechanism to therapeutics

SUPPLEMENTARY MATERIALS

Supplementary Table 1: Mutant p53 induction/repression of genes involved in metabolism or nuclear/cytoplasmic localization. See Supplementary_Table_1
