# Supplementary material for: p53 and metabolism: from mechanism to therapeutics
Source: Oncotarget. 2018 May 4;9(34):23780–823. doi: 10.18632/oncotarget.25267 (PMC5955117; doi:10.18632/oncotarget.25267)
Supplement: Supplementary file 2 [file oncotarget-09-23780-s002.docx]

**Supplementary Table 1: Mutant p53 induction/repression of genes involved in metabolism or nuclear/cytoplasmic localization**

|  | HK2 | PKM2 | GLUT  1 /4 trans | PGM | MDR1 | MLL1MLL2MOZ | TIGAR | GAMT | GLS2 | POX | ALDH | NRF2 | SESN1/2 | PGC  1a | BECN1 DRAM1 ATG12 | p-AKT | Nuc | Cyto |
| --- | --- | --- | --- | --- | --- | --- | --- | --- | --- | --- | --- | --- | --- | --- | --- | --- | --- | --- |
| 53P72R |  |  |  |  |  |  |  |  |  |  |  |  |  | +  (191) |  |  |  |  |
| p53G103S | +  (178) |  |  |  |  |  |  |  |  |  |  |  |  |  |  |  |  |  |
| p53A125T |  |  |  |  |  |  |  |  |  | 0  (97) |  |  |  |  |  |  |  |  |
| p53S149F |  |  |  |  |  |  |  |  |  | 0  (97) |  |  |  |  |  |  |  |  |
| p53P151S |  |  |  |  |  |  |  |  |  |  |  |  | -  (193) |  | -  (193) |  |  |  |
| p53R175H |  | +  (179) | +  (184) |  | +  (207) |  |  | -  (182,  185) | -  (182,  185) | - (182,  185) |  |  |  |  | -  (193,194) | 0  (198) |  |  |
| p53R181E |  |  |  |  |  |  | -  (181) | -  (185) | -  (185) | -  (185) |  |  | -  (181) |  |  |  |  |  |
| p53R193H |  |  |  |  |  |  |  |  |  | 0  (97) |  |  |  |  |  |  |  |  |
| p53G245S |  |  |  | +  (186) |  |  |  |  |  |  |  |  |  |  |  |  |  |  |
| p53G245C |  |  |  |  |  |  |  |  |  |  |  |  | -  (193) |  | -  (193) |  | +  (202) | +  (202) |
| p53M246I |  |  |  |  |  |  |  |  |  |  |  |  |  | -  (190) |  |  |  |  |
| p53R248Q |  |  | +  (184) |  | +  (208) | +  (218) |  |  |  |  |  |  |  |  |  |  |  |  |
| p53R248W |  |  |  | +  (186) | +  (207) |  |  |  |  |  |  |  |  |  |  |  | +  (202) | +  (202) |
| p53R249S |  |  |  |  |  | +  (218) |  | -  (182,  185) | -  (182,  185) | -  (182,  185) |  | 0 (105) |  |  |  |  | +  (202) | +  (202) |
| p53E256G | +  (178) |  |  |  |  |  |  |  |  |  |  |  |  |  |  |  |  |  |
|  | HK2 | PKM2 | GLUT  1 /4 trans | PGM | MDR1 | MLL1MLL2MOZ | TIGAR | GAMT | GLS2 | POX | ALDH | NRF2 | SESN1/2 | PGC  1a | BECN1 DRAM1 ATG12 | p-AKT | Nuc | Cyto |
| p53G262V |  |  |  |  |  |  |  |  |  |  |  |  |  | -  (190) |  |  |  |  |
| p53R273H |  | +  (179) | +  (184) | +  (186) | +  (207) | +  (218) | -  (182) | -  (182,  185) | -  (182,  185) | -  (182,  185) |  | -  (189) | -  (194) | -  (190) | -  (194) | +  (198) |  |  |
| p53R280K |  |  |  |  |  |  |  | -  (182,  185) | - (182,  185) | - (182,  185) | -  (182) |  |  |  |  |  | +  (202) | +  (202) |
| p53D281G |  |  |  |  | +  (207) |  |  |  |  |  |  |  |  |  |  |  |  |  |
| p53R282W |  |  |  |  |  |  |  |  |  |  |  |  | -  (193) |  | -  (193) |  | +  (202) |  |
| p53E286Q |  |  |  |  |  |  |  |  |  |  |  | 0 (105) |  |  |  |  |  |  |
| p53E339K |  |  |  |  |  |  | -  (44) |  |  |  |  |  | -  (44,  185) |  |  |  |  |  |
| p53M340Q/  p53L344R |  | +  (180) |  |  |  |  |  |  |  |  |  |  |  |  |  |  |  |  |
| p53G360A |  |  |  |  |  |  | -  (44) |  |  |  |  |  | -  (44,  185) |  |  |  |  |  |
| p53S366A |  |  |  |  |  |  | -  (44) |  |  |  |  |  | -  (44,  185) |  |  |  |  |  |
| p53S378P |  |  |  |  |  |  | -  (44) |  |  |  |  |  | -  (44,  185) |  |  |  |  |  |

HK2 (Hexokinase 2); PKM2 (pyruvate kinase isoform M2); GLUT1/4 trans (GLUT1/4 translocation); PGM (Phosphoglycerate mutase 1); MDR1 (Multi-drug resistance 1); MLL1 (Lysine Methyltransferase 2A); MLL2 (Lysine Methyltransferase 2D); MOZ (Lysine Acetyltransferase 6A); TIGAR (TP53-induced glycolysis and apoptosis regulator); GAMT (Guanidinoacetate N-Methyltransferase); GLS2 (Glutaminase 2); POX (proline oxidase); ALDH (Aldehyde Dehydrogenase 4 Family Member A1-ALDH4A1); NRF2( NF-E2 related factor 2) ; SESN 1/2 (sestrin 1 and 2); PGC1a (Peroxisome proliferator-activated receptor γ Coactivator 1-α); BECN1 (beclin 1); DRAM1 (DNA Damage Regulated Autophagy Modulator 1); ATG12 (Autophagy Related 12); p-AKT (AKT phosphorylation); Nuc (nuclear localization); Cyto (cytoplasmic localization), 0-indicates no difference, - indicates repression, + indicates induction. Number in parenthesis is the reference.
